# Power spectral analysis of voltage-gated channels in neurons

**DOI:** 10.3389/fninf.2024.1472499

**Published:** 2025-01-15

**Authors:** Christophe Magnani, Lee E. Moore

**Affiliations:** Centre Borelli, Université Paris Cité, UMR 9010, CNRS, Paris, France

**Keywords:** Hodgkin–Huxley, Markov, voltage-gated ion channels, neuronal noise, admittance, quadratic sinusoidal analysis

## Abstract

This article develops a fundamental insight into the behavior of neuronal membranes, focusing on their responses to stimuli measured with power spectra in the frequency domain. It explores the use of linear and nonlinear (quadratic sinusoidal analysis) approaches to characterize neuronal function. It further delves into the random theory of internal noise of biological neurons and the use of stochastic Markov models to investigate these fluctuations. The text also discusses the origin of conductance noise and compares different power spectra for interpreting this noise. Importantly, it introduces a novel sequential chemical state model, named *p*_2_, which is more general than the Hodgkin–Huxley formulation, so that the probability for an ion channel to be open does not imply exponentiation. In particular, it is demonstrated that the *p*_2_ (without exponentiation) and *n*^4^ (with exponentiation) models can produce similar neuronal responses. A striking relationship is also shown between fluctuation and quadratic power spectra, suggesting that voltage-dependent random mechanisms can have a significant impact on deterministic nonlinear responses, themselves known to have a crucial role in the generation of action potentials in biological neural networks.

## 1 Introduction

The purpose of this article is to elucidate the fundamental behavior of excitable neuronal membranes by using recent methods in the frequency domain. Since the historical work of the French mathematician and physicist Fourier (1822) in The Analytical Theory of Heat, it is known that many kinds of signals admit a dual representation either as a real valued function *u*(*t*) of the time variable *t* or as a complex valued function û(ω) of the frequency variable ω where û is the Fourier transform of *u*. Using this approach, individual excitable cells usually studied by their responses to constant stimuli can also be investigated by their responses to multi-sinusoidal stimuli over a broad frequency range.

With the current clamp technique, the reactions of individual cells to current stimuli generally show threshold impulses of the membrane potential that provide the means for signal transmission throughout the nervous system. Precisely because of this threshold property, it is difficult to determine the ionic currents underlying those action potential impulses. A major technical advance in measuring neuronal properties occurred with the advent of the voltage clamp technique (Bear et al., 2016), which was first invented by Cole and Marmont (1949) and later exploited by Hodgkin and Huxley (1952) who developed the Hodgkin–Huxley (HH) equations that have become the gold standard for most neuronal models in real time simulations. With the voltage clamp, a retroactive electronic device controls the membrane potential such that the neuronal properties can be quantitatively determined from the measured current elicited by a change in the potential.

Both current clamp and voltage clamp techniques can be used in the frequency domain to characterize neuronal function. A typical approach considers linear analysis by calculating impedance and admittance, as described by Mauro (1970) for the squid giant axon. However, neuronal behavior is fundamentally nonlinear due to the voltage dependence of most ionic channels (for instance potassium or sodium). Quadratic sinusoidal analysis (QSA) is a recent method developed by Magnani and Moore (2011) that provides a fundamental insight of the linear and quadratic neuronal behaviors using matrix calculus in the frequency domain. Concrete applications have been done with neurons involved in the oculo-vestibular integrator (Magnani et al., 2013) as well as with neurons of the medial entorhinal cortex which are part of the grid cell network (Magnani et al., 2014). These linear and nonlinear approaches in the frequency domain are much more efficient and concise than time domain methods for extracting stationary and dynamic features from neurons by slightly perturbing them with multi-sinusoidal signals around a steady state.

Although such a smooth deterministic description by frequency waves is able to capture fundamental properties of the neuronal function, biological neurons are significantly perturbed by internal noise. Among the different kinds of noise sources described by Stevens (1972), conductance fluctuations reflect the stochastic nature of ionic channels at the microscopic level. For this reason, stochastic Markov models have been used in this article to investigate the intrinsic fluctuations and their relationships to the complicated nonlinear behavior of neurons. This approach extends QSA analysis with stochastic Markov simulations and compares the power spectra of linear, quadratic and stochastic neuronal processes.

Some of the earliest measurements of membrane voltage noise were done on the node of Ranvier by Verveen and Derksen (1968) who suggested that the opening and closing of ionic channels lead to voltage fluctuations. Later, voltage clamp measurements on squid axons suggested that current channel noise was filtered by the axonal membrane that can be measured as a voltage power spectrum. Extensive measurements have been made of spontaneous fluctuations under both current and voltage clamp conditions along with linear impedance analysis to assess the basis of the measured noise power spectrum (Fishman et al., 1983; Conti and Wanke, 1975; Conti, 1984; Poussart, 1969; Poussart and Ganguly, 1977).

An early and perceptive paper by Stevens (1972) showed that the Hodgkin–Huxley equations themselves provide two different interpretations of the origin of conductance noise, namely the opening and closing of whole conductance channel whose opening is controlled by multiple gating particles (*n*, *m* or *h*), or alternatively, fluctuations of individual gating particles. The first case involves probability and correlation functions having multiple terms. For the potassium conductance where $g_{\text{K}}=g_{\text{K}}^{\text{max}}n^{4}$, this leads to conductance noise power spectra consisting of four Lorentzian terms. In the second case, the Hodgkin–Huxley equations are linearized and the potassium conductance power spectrum consists of a single term related to the potassium channel time constant. In the first case, the nonlinear properties of the channel (*n*^4^) would be involved in the origin of the spontaneous fluctuation, while in the second case, the linearized impedance (*n*) would likely be a good predictor of the voltage noise.

The measured squid axon spontaneous noise and the nonlinear power spectra of the Hodgkin–Huxley model both show resonance in the voltage measurements and non-resonating Lorentzian functions under voltage clamp. There is strong experimental and theoretical evidence that the spontaneous conductance noise cannot be predicted by the linear response from the same axon (Fishman et al., 1983; Poussart, 1969).

With the advent of measurements of single ionic channels in neuronal membranes, many of the detailed properties of different ionic channels as well as their macromolecular basis have dominated excitable membrane research. These findings have stimulated the development of stochastic ionic conductance models, again using the Hodgkin–Huxley equations as a fundamental basis. The gold standard method to simulate the stochastic behavior appears to be Markov models which provide fluctuation noise power spectra identical to the first Hodgkin–Huxley interpretation by Stevens discussed above. Numerous papers have derived stochastic fluctuation power spectra based on this nonlinear character (O'Donnell and van Rossum, 2014; Goldwyn and Shea-Brown, 2011, for instance). In addition to squid axon, measurements from nodes of Ranvier (Conti and Wanke, 1975; Conti, 1984; Sigworth, 1980; Elinder et al., 2001) and other preparations are consistent with the nonlinear origin of ionic power spectra. The interpretation, namely that certain nonlinear properties can show a probabilistic or stochastic character, suggests that they are involved in the fundamental origins of spontaneous channel noise in neurons. Since this clearly indicates that the stochastic behavior of excitable membranes is nonlinear, it is useful to quantitatively compare the power spectrum content of the nonlinear QSA responses with the corresponding simulations of Markov models. This will be done rigorously from the Hodgkin–Huxley equations described in the methods.

In the methods section, the Hodgkin–Huxley model is briefly reviewed. Linear analysis in the frequency domain is introduced based on the work of Mauro (1970). The quadratic sinusoidal analysis (QSA) is introduced in two ways, first the basic theory as described by Magnani and Moore (2011) and second, a new algorithm of frequency averaging to calculate power spectra. Fluctuation simulations are done with Markov models based on the work of Goldwyn et al. (2011) and Goldwyn and Shea-Brown (2011). The theoretical expressions for fluctuation power spectra are derived from the work of O'Donnell and van Rossum (2014).

In the results section, the exponentiation of the potassium *n*^4^ model is reduced to the minimal degree of nonlinearity *n*^2^. Then, a novel sequential chemical state model named *p*_2_ is introduced as a generalization of the *n*^2^ model without exponentiation. Linear analysis, fluctuation simulations and theoretical power spectra are applied to the *p*_2_ model by adapting the previous methods. Linear and quadratic functions are compared to neuronal fluctuations by adapting the previous methods. The *p*_2_ and *n*^4^ models are compared and it is demonstrated that they can produce similar neuronal responses. Remarkably, it is illustrated how the fluctuation-dissipation theorem can be violated at depolarized membrane potentials. The time constants of the *p*_2_ and *n*^4^ models seem more consistent with stochastic analysis than linear analysis.

Finally, the discussion section deals with the origin of fluctuations in the nonlinear neuronal responses. Surprisingly, simulations show that, for certain stimulus amplitudes and membrane surfaces, random voltage-dependent fluctuations can significantly modify deterministic nonlinear responses.

## 2 Methods

### 2.1 Hodgkin–Huxley model

The standard Hodgkin–Huxley model was originally proposed by Hodgkin and Huxley (1952) to describe the initiation and propagation of action potentials in the squid giant axon based on voltage clamp experiments. In this model, the current across the lipid bilayer is defined by


$$C_{m}\frac{dV}{dt}=I-I_{\text{L}}-I_{\text{K}}-I_{\text{Na}}$$

where *C*_*m*_ is the membrane capacitance, *V* is the membrane potential, *I* is the total membrane current, *I*_L_ is the leak current, *I*_K_ is the current through potassium ion channels and *I*_Na_ is the current through sodium ion channels.

The ionic currents are expressed with conductances


$$\{\begin{array}{l} \;I_{\text{L}}=g_{\text{L}}\left(V-V_{\text{L}}\right)\\ \;I_{\text{K}}=g_{\text{K}}n^{4}\left(V-V_{\text{K}}\right)\\ I_{\text{Na}}=g_{\text{Na}}m^{3}h\left(V-V_{\text{Na}}\right) \end{array}$$

where *g*_L_ is the leak conductance, *g*_K_ is the maximum potassium conductance and *g*_Na_ is the maximum sodium conductance. The constants *V*_L_, *V*_K_ and *V*_Na_ are reversal potentials for leak, potassium ion channel and sodium ion channel respectively. The gating variables *n*, *m* and *h* represent potassium channel activation, sodium channel activation and sodium channel inactivation respectively, their values are constrained between 0 and 1.

The kinetics of gating variables satisfies the following first order differential equations determined by pairs of rate constants α_*i*_(*V*) and β_*i*_(*V*)


$$\{\begin{array}{l} \;\frac{dn}{dt}=\alpha_{n}\left(V\right)\left(1-n\right)-\beta_{n}\left(V\right)n\\ \frac{dm}{dt}=\alpha_{m}\left(V\right)\left(1-m\right)-\beta_{m}\left(V\right)m\\ \;\frac{dh}{dt}=\alpha_{h}\left(V\right)\left(1-h\right)-\beta_{h}\left(V\right)h \end{array}$$

At the steady state corresponding to a membrane potential level *V*_0_, the time derivatives vanish with constants


$$\{\begin{array}{l} \;n_{\infty }\left(V_{0}\right)=\alpha_{n}\left(V_{0}\right)\tau_{n}\left(V_{0}\right)\\ m_{\infty }\left(V_{0}\right)=\alpha_{m}\left(V_{0}\right)\tau_{m}\left(V_{0}\right)\\ \;h_{\infty }\left(V_{0}\right)=\alpha_{h}\left(V_{0}\right)\tau_{h}\left(V_{0}\right) \end{array}$$

where


$$\tau_{n}={\left(\alpha_{n}+\beta_{n}\right)}^{-1},\;\tau_{m}={\left(\alpha_{m}+\beta_{m}\right)}^{-1},\;\tau_{h}={\left(\alpha_{h}+\beta_{h}\right)}^{-1}$$

### 2.2 Linear analysis

Cole (1941) was among the first to use the frequency domain to investigate neurons and suggested that the linear response of the axon membrane could be modeled with equivalent circuit elements such as inductances, capacitances and resistances. Hodgkin and Huxley also showed that their findings were consistent with the potassium conductance being described as an inductive reactance. Later, a mathematical equivalence between nonlinear conductance Hodgkin–Huxley models and electrical circuits was developed by Mauro (1970) and experimentally confirmed on the squid axon.

In papers published by Mauro (1970) and Fishman et al. (1977), the linearization of the Hodgkin–Huxley equations in the frequency domain is obtained for small perturbations at steady state. More precisely, let *X* = (*V, n, m, h*) be a dynamic state, *X*_0_ = (*V*_0_, *n*_0_, *m*_0_, *h*_0_) a steady state and $\frac{dX}{dt}=F\left(X\right)$ the system of differential equations. Clearly, *n*_0_ = *n*_∞_(*V*_0_), *m*_0_ = *m*_∞_(*V*_0_) and *h*_0_ = *h*_∞_(*V*_0_) where *V*_0_ can be controlled directly in voltage clamp or indirectly in current clamp. Then, differential calculus linearizes the system for a small perturbation steady state


$$\delta F=F\left(X\right)-F\left(X_{0}\right)\sim \delta X^{T}DF\left(X_{0}\right)$$

It follows linearization of the kinetic equations, for example for the potassium variable


$$\delta \frac{dn}{dt}=\frac{d\alpha_{n}}{dV}\delta V-\alpha_{n}\delta n-n_{0}\frac{d\alpha_{n}}{dV}\delta V-\beta_{n}\delta n-n_{0}\frac{d\beta_{n}}{dV}\delta V$$

This leads to the first order linear differential equation


$$\frac{d\delta n}{dt}+\left(\alpha_{n}+\beta_{n}\right)\delta n=\left[\frac{d\alpha_{n}}{dV}-n_{0}\frac{d\left(\alpha_{n}+\beta_{n}\right)}{dV}\right]\delta V$$

Applying the Fourier transform


$$i\omega \hat{\delta n}+\left(\alpha_{n}+\beta_{n}\right)\hat{\delta n}=\left[\frac{d\alpha_{n}}{dV}-n_{0}\frac{d\left(\alpha_{n}+\beta_{n}\right)}{dV}\right]\hat{\delta V}$$

This can be solved in the frequency domain as


$$\hat{\delta n}=\frac{\frac{d\alpha_{n}}{dV}-n_{0}\frac{d\left(\alpha_{n}+\beta_{n}\right)}{dV}}{i\omega +\alpha_{n}+\beta_{n}}\hat{\delta V}$$

and similarly for $\hat{\delta m}$ and $\hat{\delta h}$. In order to simplify notations, this fraction will be denoted as $\hat{D_{n}}$, $\hat{D_{m}}$ and $\hat{D_{h}}$ respectively.

Similarly, linearization of the ionic currents is given by


$$\{\begin{array}{l} \;\delta I_{\text{L}}=g_{\text{L}}\delta V\\ \;\delta I_{\text{K}}=4g_{\text{K}}n_{0}^{3}\left(V_{0}-V_{\text{K}}\right)\delta n+g_{\text{K}}n_{0}^{4}\delta V\\ \delta I_{\text{Na}}=3g_{\text{Na}}m_{0}^{2}h_{0}\left(V_{0}-V_{\text{Na}}\right)\delta m+g_{\text{Na}}m_{0}^{3}\left(V_{0}-V_{\text{Na}}\right)\delta h\\ \;+g_{\text{Na}}m_{0}^{3}h_{0}\delta V \end{array}$$

which can be solved in the frequency domain as


$$\{\begin{array}{l} \;\hat{\delta I_{\text{L}}}=g_{\text{L}}\hat{\delta V}\\ \;\hat{\delta I_{\text{K}}}=g_{\text{K}}\left[4n_{0}^{3}\left(V_{0}-V_{\text{K}}\right)\hat{D_{n}}+n_{0}^{4}\right]\hat{\delta V}\\ \hat{\delta I_{\text{Na}}}=g_{\text{Na}}[3m_{0}^{2}h_{0}\left(V_{0}-V_{\text{Na}}\right)\hat{D_{m}}+m_{0}^{3}\left(V_{0}-V_{\text{Na}}\right)\hat{D_{h}}\\ \;+m_{0}^{3}h_{0}]\hat{\delta V} \end{array}$$

Similarly, linearization of the current across the lipid bilayer is given by


$$C_{m}\frac{d\delta V}{dt}=\delta I-\delta I_{\text{L}}-\delta I_{\text{K}}-\delta I_{\text{Na}}$$

or equivalently in the frequency domain


$$C_{m}i\omega \hat{\delta V}=\hat{\delta I}-\hat{\delta I_{\text{L}}}-\hat{\delta I_{\text{K}}}-\hat{\delta I_{\text{Na}}}$$

The linearized response current $\hat{\delta I}$ to the linearized stimulus voltage $\hat{\delta V}$ is characterized by the admittance


$$\hat{Y}=\frac{\hat{\delta I}}{\hat{\delta V}}$$

(1) $$\begin{array}{l} \hat{Y}=C_{m}i\omega \\ \;+g_{\text{L}}\\ \;+g_{\text{K}}\left[4n_{0}^{3}\left(V_{0}-V_{\text{K}}\right)\hat{D_{n}}+n_{0}^{4}\right]\\ \;+g_{\text{Na}}\left[3m_{0}^{2}h_{0}\left(V_{0}-V_{\text{Na}}\right)\hat{D_{m}}+m_{0}^{3}\left(V_{0}-V_{\text{Na}}\right)\hat{D_{h}}+m_{0}^{3}h_{0}\right] \end{array}$$

Equivalently, the impedance is defined as


(2) $$\begin{array}{l} \hat{Z}=\frac{1}{\hat{Y}} \end{array}$$

Note that the frequency *f* in Hz is interchangeable with angular frequency ω = 2π*f*, both are used in this article depending on the context.

**Figure 1:**
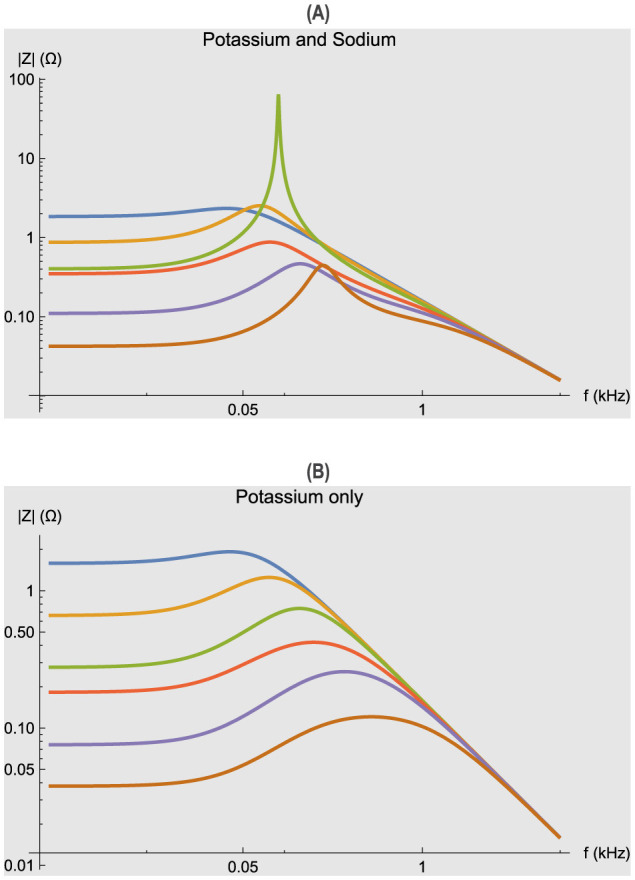
Impedance functions for the Hodgkin–Huxley model. Different membrane potential displacements *V*_0_ were applied from the resting potential of zero. Curves from top to bottom at lowest frequencies: –5, 0, 5, 10.2, 15.2, 25.2 in mV. Abscissa: frequency *f* in kHz (logarithmic scale). Ordinate: magnitude |Ẑ(*f, V*_0_)| in Ω (logarithmic scale). Parameters of the simulation are given in Table 1. **(A)** Simulations with potassium and sodium conductances. **(B)** Simulations with potassium conductance only (*g*_Na_ = 0).

One of the most striking features of neurons and their models is the presence of an impedance resonance at certain membrane potentials *V*_0_. This is illustrated for the voltage clamped Hodgkin–Huxley model especially by the two amplitude peaks at 5 and 25.2 mV depolarizations shown in Figure 1A. The plots are superimposed magnitudes |Ẑ(*f, V*_0_)| of the Equation 2, which is identical to a small signal sinusoidal stimulus of the full nonlinear Hodgkin–Huxley equations. The simulation results are similar to actual measurements on squid axons independent of the electrode properties.

Although impedance resonance is a linear property of the Hodgkin–Huxley equations, it is due to the voltage dependence of the steady state values of the ionic conductances. In particular, $\frac{dn_{\infty }}{dv}>0$ for the potassium conductance [i.e. *n*_∞_(*V*_0_) does increase with depolarizing levels *V*_0_]. In Equation 1, the potassium term in *g*_K_ was shown to be equivalent to an inductive reactance by Mauro (1970). Similarly the sodium term *g*_Na_ can be described by other circuit elements. Thus, the Hodgkin–Huxley model or any excitable cell can be analyzed by a piecewise linear analysis at different membrane potentials as shown in Figure 1A. Data collected in this manner over a range of membrane potentials allows one to determine the voltage dependence of the active conductances in addition to passive properties, and in turn construct a system of nonlinear differential equations for a particular model. Thus, a further advantage of frequency domain measurements is that model discrimination using parameter estimation can be more accurate if both real time and impedance results are used, as shown by Murphey et al. (1995).

Since neurons are composed of a minimum of two conductances, inward sodium or calcium currents and various outward potassium and other currents, it is useful to consider their individual contributions to the frequency domain behavior. This paper is focused on the potassium conductance as a model of any of the individual conductances, thus *g*_Na_ = 0. Figure 1B illustrates one broad impedance resonance maximum for the Hodgkin–Huxley potassium conductance alone, which shifts to higher frequencies with increasing depolarizations. In contrast, the impedances of Figure 1A have sharper resonances and two peaks (5 and 25.2 mV) clearly due to the presence of the sodium conductance in conjunction with potassium. Thus, one useful aspect of this analysis is that the number of resonance peaks can give an indication of the minimum number of active conductance processes present.

### 2.3 Quadratric sinusoidal analysis

Biological neurons and their models are fundamentally nonlinear systems that sometimes significantly contradict the linear superposition principle. System identification methods such as Volterra and Wiener series have been widely used to characterize nonlinear neuronal functions. In the pioneering work of Marmarelis and Naka (1972), Wiener theory was applied to predict the nonlinear behavior of a neuron chain in the catfish retina. Unfortunately, it is practically difficult and time-consuming to calculate the kernel coefficients of the Volterra and Wiener series, as these methods generally require extensive data analysis, analogous to averaging a small signal from extraneous noise.

In general, the use of random broad band stimuli similar to a white noise is time consuming, even for linear methods requiring averaging over experiments. However, significant reduction of experimental time is possible if a pseudo random stimulus containing a limited number of frequencies is applied to the preparation (Fishman et al., 1977; Poussart, 1969). Responses to such stimuli have an excellent signal to noise ratio and little or no averaging is necessary when measuring the impedance of a single cell.

In the case of nonlinear systems, multi-sinusoidal stimuli can be used to precisely measure deterministic linear and quadratic responses, provided that generated output frequencies do not overlap at first and second orders. For example, input frequencies *f*_1_ = 1, *f*_2_ = 2, *f*_3_ = 3, *f*_4_ = 4 (in Hz) generate unwanted overlaps because *f*_1_ + *f*_2_ = *f*_3_ or *f*_1_ + *f*_4_ = *f*_2_ + *f*_3_. Multi-sinusoidal stimuli with selected frequencies to avoid overlap at first and second orders were used by Magnani and Moore (2011) to study subthreshold neuronal responses, as well as previously by Victor and Shapley (1980) to study cat retinal ganglion cells.

Such non-overlapping multi-sinusoidal stimuli must have sufficiently large amplitudes to elicit both linear and quadratic responses, while remaining sufficiently small to avoid higher order contamination. The quadratic sinusoidal analysis, termed QSA, was introduced by Magnani and Moore (2011) to provide a flexible way to capture the linear and quadratic neuronal functions at subthreshold membrane potentials, as well as to compare biological experiments with theoretical models (Magnani et al., 2013, 2014). In particular, the subthreshold membrane potential just below the threshold is fundamentally a nonlinear process which is critically involved in action potential generation.

It is well known that a sinusoidal signal can be expressed as a sum of complex exponentials in Fourier analysis, for example :


$$\cos\left(\omega t\right)=\frac{1}{2}e^{i\omega t}+\frac{1}{2}e^{-i\omega t}$$

where *i*^2^ = −1 and ω is the angular frequency. More generally, a multi-sinusoidal signal of *N* frequencies can be expressed as a sum of complex exponentials


$$x\left(t\right)=\underset{k\in \text{Γ}}{\sum }x_{k}e^{i\omega_{k}t}$$

where Γ = {−*N*, ⋯ , −1, +1, ⋯ , +*N*} enumerates integers between −*N* and +*N* (zero excluded), *x*_*k*_ are complex Fourier coefficients such that $x_{-k}=\bar{x_{k}}$ and ω_*k*_ are angular frequencies such that ω_−*k*_ = −ω_*k*_. The duration *T* (s) of the experiment determines the lowest frequency $\frac{1}{T}$ (Hertz). The angular frequencies are defined by integer multiples of the lowest frequency, that is to say $\omega_{k}=2\pi \frac{n_{k}}{T}$ where *n*_*k*_ are integers such that *n*_−*k*_ = −*n*_*k*_. Each Fourier coefficient is a complex number that can be decomposed as $x_{k}=|x_{k}|e^{i\theta_{k}}$ where |*x*_*k*_| is the amplitude (nonnegative real number) and θ_*k*_ is the phase (between 0 and 2π). It is good practice to randomize the phases of multi-sinusoidal stimuli to avoid biases in neuronal responses.

Such a multi-sinusoidal stimulus *x*(*t*) can be applied to neuronal cells in voltage clamp and current clamp experiments. Assuming that quality criteria for the QSA method are satisfied (Magnani and Moore, 2011), the output signal measured by the electrode can be decomposed as


$$y\left(t\right)=y_{0}+y_{1}\left(t\right)+y_{2}\left(t\right)$$

where *y*_0_ is the DC component, *y*_1_(*t*) is the linear component and *y*_2_(*t*) is the quadratic component. More specifically


(3) $$\begin{array}{l} y\left(t\right)=y_{0}+\underset{k\in \text{Γ}}{\sum }l_{k}x_{k}e^{i\omega_{k}t}+\underset{i,j\in \text{Γ}}{\sum }b_{i,j}x_{i}e^{i\omega_{i}t}x_{j}e^{i\omega_{j}t} \end{array}$$

where *l*_*k*_ and *b*_*i, j*_ are complex numbers characterizing the neuronal response to the stimulus, by similarity with impedance or admittance. By convention, *b*_−*k, k*_ = 0 for all *k* ∈ Γ so that all DC components are encoded in *y*_0_.

The QSA theory is based on a vector representation of multi-sinusoidal signals in an orthonormal vector basis {_***e*_*k*_**}*k* ∈ Γ_ representing the complex exponentials ${\left\{e^{i\omega_{k}t}\right\}}_{k\in \Gamma }$. In this way, the multi-sinusoidal stimulus *x*(*t*) is encoded as a time independent vector


$$\text{x}=\underset{k\in \text{Γ}}{\sum }x_{k}{\text{e}}_{k}$$

The corresponding time dependent vectors are defined by


$${\text{x}}_{t}=\underset{k\in \text{Γ}}{\sum }x_{k}e^{i\omega_{k}t}{\text{e}}_{k}$$

Putting the coefficients *l*_*k*_ in a row matrix **L** and the coefficients *b*_*i, j*_ in a square matrix **B**, Equation 3 can be reformulated with linear algebra as


(4) $$\begin{array}{l} y\left(t\right)=y_{0}+\text{L}{\text{x}}_{t}+{\text{x}}_{t}^{T}\text{B}{\text{x}}_{t} \end{array}$$

By noticing that


$$\begin{array}{ll} \underset{i,j\in \text{Γ}}{\sum }b_{i,j}x_{i}e^{i\omega_{i}t}x_{j}e^{i\omega_{j}t} & =\underset{i,j\in \text{Γ}}{\sum }b_{-i,j}\bar{x_{i}e^{i\omega_{i}t}}x_{j}e^{i\omega_{j}t} \end{array}$$

we are led to define the QSA matrix **Q** by


$$Q_{i,j}=B_{-i,j}$$

so that


$$y\left(t\right)=y_{0}+\text{L}{\text{x}}_{t}+{\bar{\text{x}}}_{t}^{T}\text{Q}{\text{x}}_{t}$$

Remarkably, the QSA matrix **Q** is Hermitian (Magnani and Moore, 2011), which means that


$${\bar{\text{Q}}}^{T}=\text{Q}$$

This algebraic approach has been widely used in modern physics, especially in quantum physics where Hermitian operators represent physical observables. In particular, the eigenvalues of the QSA matrix are real numbers which form a spectrum that can be interpreted as a signature of the quadratic neuronal function.

The most glaring property of the quadratic neuronal response is the generation of second order frequencies that are not present in the stimulus. More precisely, Equation 3 shows that the linear components $l_{k}x_{k}e^{i\omega_{k}t}$ contain only the stimulus frequencies ω_*k*_, while the quadratic components $b_{i,j}x_{i}e^{i\omega_{i}t}x_{j}e^{i\omega_{j}t}=b_{i,j}x_{i}x_{j}e^{i\left(\omega_{i}+\omega_{j}\right)t}$ contain new frequencies ω_*i*_ + ω_*j*_ that are not present in the stimulus in the absence of overlap. The row matrix **L** and the QSA matrix **Q** can be interpreted as linear and quadratic filters respectively, by extension of the impedance or admittance concept.

### 2.4 Multi-sinusoidal power spectra

The concept of power spectrum is useful in statistical analysis, both for signal processing and for stochastic processes, so it is natural to use it in this paper. The power spectrum of a multi-sinusoidal signal *u*(*t*) is given by |û(ω)|^2^ where û is the Fourier transform of *u* and ω is the frequency variable. The power spectrum can be averaged over a set of measurements.

For a single measurement of the output *y*(*t*) in response to a stimulus *x*(*t*) with fundamental frequencies |ω_*k*_|, the multi-sinusoidal power spectra cover the positive frequencies of linear and quadratic analyses:

- Linear power spectrum at fundamental frequencies:
  $$S_{L}\left(|\omega_{k}|\right)=|{\hat{y}}_{1}\left(|\omega_{k}|\right){|}^{2}$$
- Quadratic power spectrum at frequency doubling:
  $$S_{D}\left(2|\omega_{k}|\right)=|{\hat{y}}_{2}\left(2|\omega_{k}|\right){|}^{2}$$
- Quadratic power spectrum at frequency sums for |ω_*i*_| ≠ |ω_*j*_|:
  $$S_{P}\left(|\omega_{i}|+|\omega_{j}|\right)=|{\hat{y}}_{2}\left(|\omega_{i}|+|\omega_{j}|\right){|}^{2}$$
- Quadratic power spectrum at frequency differences for |ω_*i*_| ≠ |ω_*j*_|:
  $$S_{M}\left(||\omega_{i}|-|\omega_{j}||\right)=|{\hat{y}}_{2}\left(||\omega_{i}|-|\omega_{j}||\right){|}^{2}$$

The quadratic power spectra are indexed by second order frequencies, which are not stimulus frequencies because they were chosen without overlap. Thus, it would be convenient to have also a quadratic power spectrum indexed by fundamental frequencies as an alternative representation. To this end, a function similar to the “R summation function” of Magnani et al. (2013) is introduced in a different way below, computing the mean squared quadratic output by matrix columns:


$$S_{R}\left(\omega_{j}\right)=\frac{1}{2N}\underset{i\in \text{Γ}}{\sum }|Q_{i,j}\bar{x_{i}}x_{j}{|}^{2}$$

Although these multi-sinusoidal power spectra reflect exact neuronal responses, they are not accurate because they are defined over a small set of non-overlapping frequencies. Therefore, it is necessary to average multiple measurements to increase the accuracy of the power spectra.

To perform this averaging, a set of *M* stimuli *x*^(*m*)^(*t*) is generated with sets of non-overlapping random frequencies Ω^(*m*)^ for *m* = 1, …, *M*. Each set of first order frequencies Ω^(*m*)^ determines a set of second order frequencies Ξ^(*m*)^. The global sets of first and second order frequencies are defined by merging the individual sets, respectively


$$\{\begin{array}{l} \Omega =\overset{M}{\underset{m=1}{⋃}}\Omega^{\left(m\right)}\\ \;\Xi =\overset{M}{\underset{m=1}{⋃}}\Xi^{\left(m\right)} \end{array}$$

Importantly, different individual sets of the same order can share frequencies, so it is necessary to count redundancies


$$\{\begin{array}{l} N_{\Omega }\left(\omega \right)=\#\left\{m|\omega \in \Omega^{\left(m\right)}\right\}\\ \;N_{\Xi }\left(\xi \right)=\#\left\{m|\xi \in \Xi^{\left(m\right)}\right\} \end{array}$$

where # indicates the number of elements in a set.

Fourier analysis of the measured linear responses $y_{1}^{\left(m\right)}\left(t\right)$ yields output sets ${\hat{y}}_{1}^{\left(m\right)}\left(\omega \right)$ defined at the first order frequencies ω ∈ Ω^(*m*)^ and zero elsewhere. This allows to calculate the averaged linear power spectrum for ω ∈ Ω


$$S_{L}\left(\omega \right)=\frac{1}{N_{\Omega }\left(\omega \right)}\overset{M}{\underset{m=1}{\sum }}|{\hat{y}}_{1}^{\left(m\right)}\left(\omega \right){|}^{2}$$

where ω ∈ Ω implies $N_{\Omega }\left(\omega \right)\geq 1$ so the denominator is not zero.

Fourier analysis of the measured quadratic responses $y_{2}^{\left(m\right)}\left(t\right)$ yields output sets ${\hat{y}}_{2}^{\left(m\right)}\left(\xi \right)$ defined at the second order frequencies ξ ∈ Ξ^(*m*)^ and zero elsewhere. This allows to calculate the averaged quadratic power spectrum for ξ ∈ Ξ


$$S_{2}\left(\xi \right)=\frac{1}{N_{\Xi }\left(\xi \right)}\overset{M}{\underset{m=1}{\sum }}|{\hat{y}}_{2}^{\left(m\right)}\left(\xi \right){|}^{2}$$

where ξ ∈ Ξ implies N(ξ)≥1 so the denominator is not zero. The partial quadratic power spectra *S*_*D*_, *S*_*P*_, *S*_*M*_ can be calculated using the same method.

The power spectra *S*_*R*_ are calculated on first order frequencies using the same method for ω ∈ Ω


$$S_{R}\left(\omega \right)=\frac{1}{N_{\Omega }\left(\omega \right)}\overset{M}{\underset{m=1}{\sum }}S_{R}^{\left(m\right)}\left(\omega \right)$$

In particular, ω ∈ Ω implies $N_{\Omega }\left(\omega \right)\geq 1$ so the denominator is not zero.

It should be noted that although different sets Ω^(*m*)^ and Ξ^(*m*)^ may share frequencies, such post-result frequency redundancy is not frequency overlap, with each individual measurement *y*^(*m*)^(*t*) being the response to a stimulus without overlap.

### 2.5 Stochastic Markov simulations

Although the Hodgkin–Huxley model is empirical, it has led to various biophysical interpretations. The potassium ion channel is generally considered to have four independent identical subunits, each characterized by a two-state process


$$0\overset{\alpha }{\underset{\beta }{⇌}}1$$

where α_*n*_(*V*) and β_*n*_(*V*) are voltage-dependent transition rates given in the Hodgkin–Huxley model. All four subunits must be open for the channel to be open. If *n* denotes the gating variable for subunit activation, the total potassium conductance is proportional to *n*^4^.

This four-subunits interpretation suggests molecular-scale conformational changes that exceed the accuracy of the Hodgkin–Huxley model. However, this concept provides a theoretical interpretation of the fundamental nonlinearity of the neuron that is interesting for exploring equivalent forms of the Hodgkin–Huxley model.

The original Hodgkin–Huxley model is nonlinear in two ways: one is the use of an exponentiation for the gating variable, such as *n*^4^; the other is the voltage dependence of the rate constants. In the first case, an exponentiation is used to describe the delay in current response observed after a step change in membrane potential. In the second case, the use of the voltage clamp at a constant membrane potential *V*_0_ has the effect of linearizing the differential equation $\frac{dn}{dt}=\alpha_{n}\left(V_{0}\right)\left(1-n\right)-\beta_{n}\left(V_{0}\right)n$ since the rate constants α_*n*_(*V*_0_) and β_*n*_(*V*_0_) become constants.

The two-state process can be extended to multiple sequential states typical of higher order chemical relaxation models. More precisely, the four independent identical subunits of the potassium ion channel can be modeled as a five-state Markov chain


(5) $$\begin{array}{l} 0\overset{4\alpha_{n}}{\underset{\beta_{n}}{⇌}}1\overset{3\alpha_{n}}{\underset{2\beta_{n}}{⇌}}2\overset{2\alpha_{n}}{\underset{3\beta_{n}}{⇌}}3\overset{\alpha_{n}}{\underset{4\beta_{n}}{⇌}}4 \end{array}$$

where each state 0, 1, 2, 3, 4 corresponds to the number of open subunits at a given time. Each channel has the probability *p*_*k*_(*t*) to be in state *k*. In particular, 1 = *p*_0_ + *p*_1_ + *p*_2_ + *p*_3_ + *p*_4_. The channel is open when all four subunits are open, which corresponds to state 4 with probability *p*_4_. When the system is in the state *k*, there are *k* open subunits and (4 − *k*) closed subunits. For *k* < 4, the transition *k* → *k* + 1 opens one of the (4 − *k*) closed subunits, which corresponds to the rate (4 − *k*) α _*n*_. Similarly, for *k* > 0, the transition *k* → *k* − 1 closes one of the *k* open subunits, which corresponds to the rate *kβ*_*n*_.

More generally, arbitrary rate constants could be chosen other than integer multiples of α_*n*_ and β_*n*_. Numerous models of this type with more than two independent rate constants have been compared to the original Hodgkin–Huxley model, e.g. Vandenberg and Bezanilla (1991).

Stochastic Markov simulations were based on Gillespie (1977) and Goldwyn et al. (2011); Goldwyn and Shea-Brown (2011) considering a statistical population of *N* potassium ion channels and a time interval *t* ∈ [0;*T*] discretized by Δ*t*. Let *N*_*k*_(*t*) be the number of channels in state *k* at time *t*. In particular, *N* = *N*_0_ + *N*_1_ + *N*_2_ + *N*_3_ + *N*_4_. In the limit, for a large population, the proportion of channels in state *k* tends to the corresponding probability


$$\underset{N\to \infty }{\lim}\frac{N_{k}\left(t\right)}{N}=p_{k}\left(t\right)$$

Let Δ*N*_*k*_(*t*) be the variation of the number of channels in state *k* during Δ*t*. Let Δ*N*_*i*→*j*_(*t*) be the number of channels switching from state *i* to state *j* during Δ*t*.

To ensure validity, it will always be assumed that *p*_*k*_, *N*_*k*_, Δ*N*_*k*_, Δ*N*_*i*→*j*_ are zero for indices outside the range {0, 1, 2, 3, 4}.

The proportion of channels switching from state *k* to state *k* ± 1 during Δ*t* is determined by the transition rates


$$\{\begin{array}{l} \text{Δ}N_{k\to k+1}=\left(4-k\right)\alpha_{n}N_{k}\text{Δ}t\\ \text{Δ}N_{k\to k-1}=k\beta_{n}N_{k}\text{Δ}t \end{array}$$

Furthermore, the variation of the number of channels in state *k* during Δ*t* corresponds to the number of transitions to state *k* minus the number of transitions from state *k*. More precisely


$$\text{Δ}N_{k}=\text{Δ}N_{k-1\to k}+\text{Δ}N_{k+1\to k}-\text{Δ}N_{k\to k-1}-\text{Δ}N_{k\to k+1}$$

Replacing expressions


$$\begin{array}{r} \text{Δ}N_{k}=\left(5-k\right)\alpha_{n}N_{k-1}\text{Δ}t+\left(k+1\right)\beta_{n}N_{k+1}\text{Δ}t-k\beta_{n}N_{k}\text{Δ}t\\ -\left(4-k\right)\alpha_{n}N_{k}\text{Δ}t \end{array}$$

Dividing by *N* and taking the limit, a system of differential equations is obtained


$$\frac{dp_{k}}{dt}=\left(5-k\right)\alpha_{n}p_{k-1}+\left(k+1\right)\beta_{n}p_{k+1}-k\beta_{n}p_{k}-\left(4-k\right)\alpha_{n}p_{k}$$

More explicitly, this gives the master equation


$$\{\begin{array}{l} \frac{dp_{0}}{dt}=-4\alpha_{n}p_{0}+\beta_{n}p_{1}\\ \frac{dp_{1}}{dt}=4\alpha_{n}p_{0}-\left(3\alpha_{n}+\beta_{n}\right)p_{1}+2\beta_{n}p_{2}\\ \frac{dp_{2}}{dt}=3\alpha_{n}p_{1}-2\left(\alpha_{n}+\beta_{n}\right)p_{2}+3\beta_{n}p_{3}\\ \frac{dp_{3}}{dt}=2\alpha_{n}p_{2}-\left(\alpha_{n}+3\beta_{n}\right)p_{3}+4\beta_{n}p_{4}\\ \frac{dp_{4}}{dt}=\alpha_{n}p_{3}-4\beta_{n}p_{4} \end{array}$$

The conductance of the population of channels is calculated as *g*_K_*f* where *g*_K_ is the conductance of an individual channel and *f* is the proportion of open channels (Goldwyn et al., 2011). In the limit, for a large population, the proportion of open channels tends to the probability *p*_4_ that a channel is open, which corresponds to the deterministic conductance *g*_K_*p*_4_.

Although the master equation does not use exponentiation, the equivalence with the *n*^4^ model is discussed by Dayan and Abbott (2001). Indeed, in the Hodgkin–Huxley model, *n* is a gating variable representing the probability that a subunit is open and 1 − *n* the probability that it is closed. In state *k*, *k* of the four subunits are open and the 4 − *k* others are closed, thus $p_{k}=\left(\begin{array}{c} 4\\ k \end{array}\right)n^{k}{\left(1-n\right)}^{4-k}$. In particular, in state 4, all four subunits are open, thus $p_{4}=n^{4}$ which is consistent with the exponentiation of the Hodgkin–Huxley model.

Therefore, at a fundamental level, nonlinearities generated by neuronal responses are likely to reflect fluctuations between internal states for which the master equation is controlled by transition rates involving energy for the movement of charges, the associated Boltzmann factor is discussed by Dayan and Abbott (2001). The master equation provides a deterministic description of the probabilities of these fluctuations.

Although Markov simulations are widely used to describe ion channel noise in the Hodgkin–Huxley model, alternative methods have been proposed, offering different trade-offs between accuracy and complexity. The Langevin approach, introduced by Fox and Lu (1994) and further refined by Fox (1997), incorporates stochasticity by adding Gaussian white noise terms to the equations, making it suitable for systems with a large number of channels. Goldwyn and Shea-Brown (2011) critically evaluated such stochastic formulations, highlighting the limitations of certain approximations in accurately representing neuronal behavior. Linaro et al. (2011) developed an improved diffusion approximation that enhances the accuracy of simulations, providing a more reliable alternative for neuronal modeling. Furthermore, Baravalle et al. (2017) presented a path integral approach to the Hodgkin–Huxley model, using methods from theoretical physics to analyze stochastic dynamics in which noise processes may be influenced by neural network interactions and feedforward correlations.

The advantage of Markov models lies in their ability to explicitly represent individual states of ion channels and the stochastic transitions between them, closely reflecting microscopic behavior. Unlike traditional Hodgkin–Huxley models, they inherently capture stochastic fluctuations in channel opening and closing, as transitions are governed by probabilistic processes. This enables Markov models to account for complex behaviors, including multiple transition pathways, nonlinear kinetics, and independent inactivation or recovery processes that are challenging to address with conventional methods. Notably, Markov models establish a connection between stochastic processes and nonlinear multiplicative effects, such as $p_{4}=n^{4}$, while Langevin approaches are based on the addition of noise. Furthermore, hierarchical Markov models (Siekmann et al., 2016) allow for the inclusion of modal gating of ion channels, capturing both transitions between modes and stochastic dynamics within modes. Overall, Markov models provide a scalable and versatile method for simulating neuronal phenomena, widely recognized as a standard in the field.

### 2.6 Markov power spectra

Stochastic analysis of Markov models provides power spectra of conductance noise identical to the first interpretation of Stevens (1972) involving probability and correlation functions. Many papers have derived noise power spectra based on the nonlinear nature of the potassium channel (*n*^4^), especially O'Donnell and van Rossum (2014) which is used in this article. In addition to the squid axon, measurements on the nodes of Ranvier (Conti, 1984) are consistent with the nonlinear origin of the conductance noise power spectra. Such an interpretation that certain nonlinear properties can induce a probabilistic or stochastic character, suggests that they are involved in the fundamental origin of spontaneous fluctuations in neurons.

The conductance noise can be predicted from the Hodgkin–Huxley equations for potassium conductance as described by O'Donnell and van Rossum (2014). Their approach is explored here for further adaptation in this article.

Let *X*_*t*_ be the random variable measuring if the channel is open (*X*_*t*_ = 1) or closed (*X*_*t*_ = 0) at time *t*. Let *p*(*t*) be the probability that the channel is open at time *t*, namely *p*(*t*) = *p*(*X*_*t*_ = 1). Let *p*_∞_ be the steady state probability, which coincides with the average of *p*(*t*) over time. Actually, $p=p_{4}=n^{4}$ using the previous Markov probability notations, but the *p* notation is used here to be more general.

By definition, the autocorrelation *r*(*t*_1_, *t*_2_) characterizes the similarity of *X*_*t*_ between times *t*_1_ and *t*_2_. Denoting by **E** the expected value of a random variable


$$r\left(t_{1},t_{2}\right)=\text{E}\left[X_{t_{1}}X_{t_{2}}\right]$$

Since *X*_*t*_ only takes the values 0 or 1, the autocorrelation is given by


$$\begin{array}{l} r\left(t_{1},t_{2}\right)=\underset{i,j\in \left\{0,1\right\}}{\sum }i\cdot j\cdot p\left(X_{t_{1}}=i,X_{t_{2}}=j\right)\\ \;=p\left(X_{t_{1}}=1,X_{t_{2}}=1\right)\\ \;=p\left(X_{t_{2}}=1|X_{t_{1}}=1\right)p\left(X_{t_{1}}=1\right) \end{array}$$

or more concisely


$$r\left(t_{1},t_{2}\right)=p\left(t_{1}\right)p\left(t_{2}|t_{1}\right)$$

The autocorrelation can also be reformulated betwen times *t*_0_ and *t*_0_ + *s* to make the time lag *s* explicit


$$r\left(t_{0},t_{0}+s\right)=p\left(t_{0}\right)p\left(t_{0}+s|t_{0}\right)$$

Here, *p*(*t*_0_ + *s* ∣ *t*_0_) is the conditional probability that the channel is open at time *t*_0_ + *s* provided that it is open at time *t*_0_. Importantly, if the channel is open at time *t*_0_ then *p*(*t*_0_) = 1. In this special case, the Markov state (*p*_0_, *p*_1_, *p*_2_, *p*_3_, *p*_4_) = (0, 0, 0, 0, 1) is unique due to the constraint *p*_0_ + *p*_1_ + *p*_2_ + *p*_3_ + *p*_4_ = 1. The same reasoning is valid for other sequential models with fewer or more Markov states. Thus, the time evolution of *p*(*t*_0_ + *s* ∣ *t*_0_) from the unique state (0, 0, 0, 0, 1) between *t*_0_ and *t*_0_ + *s* is identical to the time evolution of *p*(*s* ∣ 0) from the unique state (0, 0, 0, 0, 1) between 0 and *s*, the two trajectories of the dynamical system are identical because they start from the same unique state (0, 0, 0, 0, 1) and have the same duration *s*. Therefore


$$r\left(t_{0},t_{0}+s\right)=p\left(t_{0}\right)p\left(s|0\right)$$

Averaging over the time origin *t*_0_ gives the autocorrelation as a function of the time lag


$$\begin{array}{l} r\left(s\right)={\langle r\left(t_{0},t_{0}+s\right)\rangle }_{t_{0}}\\ \;={\langle p\left(t_{0}\right)p\left(s|0\right)\rangle }_{t_{0}}\\ \;={\langle p\left(t_{0}\right)\rangle }_{t_{0}}p\left(s|0\right)\\ \;=p_{\infty }p\left(s|0\right) \end{array}$$

By definition, the autocovariance *C*(*t*_1_, *t*_2_) characterizes the similarity of *X*_*t*_ − **E**[*X*_*t*_] between times *t*_1_ and *t*_2_. Namely


$$\begin{array}{l} C\left(t_{1},t_{2}\right)=\text{E}\left[\left(X_{t_{1}}-\text{E}\left[X_{t_{1}}\right]\right)\left(X_{t_{2}}-\text{E}\left[X_{t_{2}}\right]\right)\right]\\ \;=\text{E}\left[\left(X_{t_{1}}-p_{\infty }\right)\left(X_{t_{2}}-p_{\infty }\right)\right]\\ \;=\text{E}\left[X_{t_{1}}X_{t_{2}}\right]-p_{\infty }\text{E}\left[X_{t_{1}}\right]-p_{\infty }\text{E}\left[X_{t_{2}}\right]+p_{\infty }^{2}\\ \;=r\left(t_{1},t_{2}\right)-p_{\infty }^{2} \end{array}$$

The autocovariance can also be reformulated betwen times *t*_0_ and *t*_0_ + *s* to make the time lag *s* explicit


$$C\left(t_{0},t_{0}+s\right)=r\left(t_{0},t_{0}+s\right)-p_{\infty }^{2}$$

Averaging over the time origin *t*_0_ gives the autocovariance as a function of the time lag


$$\begin{array}{l} C\left(s\right)={\langle C\left(t_{0},t_{0}+s\right)\rangle }_{t_{0}}\\ \;=r\left(s\right)-p_{\infty }^{2}\\ \;=p_{\infty }p\left(s|0\right)-p_{\infty }^{2} \end{array}$$

When the channel is open, the single-channel current is given by Ohm's law, namely *i*_K_ = γ_K_(*V* − *V*_K_) where γ_*K*_ is the single-channel conductance, *V* is the voltage imposed by voltage clamp and *V*_K_ is the reversal potential. Then, the fluctuating single-channel current is defined as *i*_K_*X*_*t*_. In particular, the fluctuating single-channel current is zero when the channel is closed and is given by Ohm's law when the channel is open. For a population of *N*_K_ channels, let $X_{t}^{n}$ be the random variables for each channel *n* = 1…*N*_K_. The total fluctuating current is


$$I_{\text{K}}\left(t\right)=\overset{N_{\text{K}}}{\underset{n=1}{\sum }}i_{\text{K}}X_{t}^{n}$$

By definition, the autocovariance *C*_*I*_K__(*t*_1_, *t*_2_) of the total fluctuating current between times *t*_1_ and *t*_2_ is given by


$$\begin{array}{l} C_{I_{\text{K}}}\left(t_{1},t_{2}\right)=\text{E}\left[\left(I_{\text{K}}\left(t_{1}\right)-\text{E}\left[I_{\text{K}}\left(t_{1}\right)\right]\right)\left(I_{\text{K}}\left(t_{2}\right)-\text{E}\left[I_{\text{K}}\left(t_{2}\right)\right]\right)\right]\\ \;=\text{E}[\left(\overset{N_{\text{K}}}{\underset{n=1}{\sum }}i_{\text{K}}X_{t_{1}}^{n}-\text{E}\left[\overset{N_{\text{K}}}{\underset{n=1}{\sum }}i_{\text{K}}X_{t_{1}}^{n}\right]\right)(\overset{N_{\text{K}}}{\underset{m=1}{\sum }}i_{\text{K}}X_{t_{2}}^{m}\\ \;-\text{E}\left[\overset{N_{\text{K}}}{\underset{m=1}{\sum }}i_{\text{K}}X_{t_{2}}^{m}])\right]\\ \;=i_{\text{K}}^{2}\text{E}[\left(\overset{N_{\text{K}}}{\underset{n=1}{\sum }}X_{t_{1}}^{n}-\overset{N_{\text{K}}}{\underset{n=1}{\sum }}\text{E}\left[X_{t_{1}}^{n}\right]\right)(\overset{N_{\text{K}}}{\underset{m=1}{\sum }}X_{t_{2}}^{m}\\ \;-\overset{N_{\text{K}}}{\underset{m=1}{\sum }}\text{E}[X_{t_{2}}^{m}])]\\ \;=i_{\text{K}}^{2}\text{E}\left[\left(\overset{N_{\text{K}}}{\underset{n=1}{\sum }}\left(X_{t_{1}}^{n}-\text{E}\left[X_{t_{1}}^{n}\right]\right)\right)\left(\overset{N_{\text{K}}}{\underset{m=1}{\sum }}\left(X_{t_{2}}^{m}-\text{E}\left[X_{t_{2}}^{m}\right]\right)\right)\right]\\ \;=i_{\text{K}}^{2}\overset{N_{\text{K}}}{\underset{n=1}{\sum }}\overset{N_{\text{K}}}{\underset{m=1}{\sum }}\text{E}\left[\left(X_{t_{1}}^{n}-p_{\infty }\right)\left(X_{t_{2}}^{m}-p_{\infty }\right)\right] \end{array}$$

It can be noticed that


$$\begin{array}{l} \text{E}\left[\left(X_{t_{1}}^{n}-p_{\infty }\right)\left(X_{t_{2}}^{m}-p_{\infty }\right)\right]=\text{E}\left[X_{t_{1}}^{n}X_{t_{2}}^{m}\right]-p_{\infty }\text{E}\left[X_{t_{1}}^{n}\right]-p_{\infty }\text{E}\left[X_{t_{2}}^{m}\right]+p_{\infty }^{2}\\ \;=\text{E}\left[X_{t_{1}}^{n}X_{t_{2}}^{m}\right]-p_{\infty }^{2} \end{array}$$

If *n* ≠ *m*, the random variables $X_{t}^{n}$ and $X_{t}^{m}$ are independent, then


$$\text{E}\left[\left(X_{t_{1}}^{n}-p_{\infty }\right)\left(X_{t_{2}}^{m}-p_{\infty }\right)\right]=\text{E}\left[X_{t_{1}}^{n}\right]\text{E}\left[X_{t_{2}}^{m}\right]-p_{\infty }^{2}=p_{\infty }p_{\infty }-p_{\infty }^{2}=0$$

If *n* = *m*, the random variables $X_{t}^{n}$ and $X_{t}^{m}$ are the same as *X*_*t*_, then


$$\text{E}\left[\left(X_{t_{1}}^{n}-p_{\infty }\right)\left(X_{t_{2}}^{m}-p_{\infty }\right)\right]=\text{E}\left[X_{t_{1}}X_{t_{2}}\right]-p_{\infty }^{2}=r\left(t_{1},t_{2}\right)-p_{\infty }^{2}=C\left(t_{1},t_{2}\right)$$

These remarks on the indices *n* and *m* allow to simplify the double sum in the autocovariance of the total fluctuating current so that


$$\begin{array}{l} C_{I_{\text{K}}}\left(t_{1},t_{2}\right)=i_{\text{K}}^{2}\overset{N_{\text{K}}}{\underset{n=1}{\sum }}C\left(t_{1},t_{2}\right)\\ \;=N_{\text{K}}i_{\text{K}}^{2}C\left(t_{1},t_{2}\right) \end{array}$$

The autocovariance can also be reformulated betwen times *t*_0_ and *t*_0_ + *s* to make the time lag *s* explicit


$$C_{I_{\text{K}}}\left(t_{0},t_{0}+s\right)=N_{\text{K}}i_{\text{K}}^{2}C\left(t_{0},t_{0}+s\right)$$

Averaging over the time origin *t*_0_ gives the autocovariance as a function of the time lag


$$\begin{array}{l} C_{I_{\text{K}}}\left(s\right)={\langle N_{\text{K}}i_{\text{K}}^{2}C\left(t_{0},t_{0}+s\right)\rangle }_{t_{0}}\\ \;=N_{\text{K}}i_{\text{K}}^{2}{\langle C\left(t_{0},t_{0}+s\right)\rangle }_{t_{0}}\\ \;=N_{\text{K}}i_{\text{K}}^{2}C\left(s\right) \end{array}$$

The Wiener–Khinchin theorem provides the power spectrum $S_{I_{\text{K}}}^{\pm }$ as the Fourier transform of the autocovariance *C*_*I*_K__, using the non-unitary Fourier transform with angular frequencies


$$S_{I_{\text{K}}}^{\pm }\left(\omega \right)=\int_{-\infty }^{\infty }C_{I_{\text{K}}}\left(s\right)e^{-i\omega s}ds$$

The autocovariance is an even function of the lag because under stationarity, the average over the time origin *t*_0_ is independent of a time lag


$$\begin{array}{l} C\left(s\right)={\langle C\left(t_{0},t_{0}+s\right)\rangle }_{t_{0}}\\ \;={\langle C\left(t_{0}-s,t_{0}\right)\rangle }_{t_{0}-s}\\ \;={\langle C\left(t_{0}-s,t_{0}\right)\rangle }_{t_{0}}\\ \;={\langle C\left(t_{0},t_{0}-s\right)\rangle }_{t_{0}}\\ \;=C\left(-s\right) \end{array}$$

Thus, the power spectrum can be decomposed into two parts


$$\begin{array}{l} S_{I_{\text{K}}}^{\pm }\left(\omega \right)=\int_{-\infty }^{0}C_{I_{\text{K}}}\left(s\right)e^{-i\omega s}ds+\int_{0}^{+\infty }C_{I_{\text{K}}}\left(s\right)e^{-i\omega s}ds\\ \;=\int_{0}^{+\infty }C_{I_{\text{K}}}\left(-s\right)e^{i\omega s}ds+\int_{0}^{+\infty }C_{I_{\text{K}}}\left(s\right)e^{-i\omega s}ds\\ \;=\int_{0}^{+\infty }C_{I_{\text{K}}}\left(s\right)\left[e^{i\omega s}+e^{-i\omega s}\right]ds\\ \;=2\int_{0}^{+\infty }C_{I_{\text{K}}}\left(s\right)\cos\left(\omega s\right)ds \end{array}$$

To follow the convention of O'Donnell and van Rossum (2014), the power spectrum *S*_*I*_K__ will be considered for positive frequencies only, i.e.


$$S_{I_{\text{K}}}\left(\omega \right)=2S_{I_{\text{K}}}^{\pm }\left(\omega \right)=4ℜ\int_{0}^{+\infty }C_{I_{\text{K}}}\left(s\right)e^{-i\omega s}ds$$

In order to calculate the Markov power spectra for the fluctuating potassium current, it is necessary to determine the autocovariance $C\left(s\right)=p_{\infty }p\left(s|0\right)-p_{\infty }^{2}$ based on the two components *p*(*s*∣0) and *p*_∞_. The kinetics of the potassium gating variable *n* is given by


$$\frac{dn}{dt}=\alpha_{n}\left(1-n\right)-\beta_{n}n$$

or equivalently


$$\frac{dn}{dt}=\frac{n_{\infty }-n}{\tau_{n}}$$

The general solution can be formulated with an exponential decay and an arbitrary initial condition *n*_0_


$$n\left(t\right)=n_{\infty }+\left(n_{0}-n_{\infty }\right)e^{-t/\tau_{n}}$$

The solution *n*(*t*∣0) is interpreted as the conditional probability that the gate is open at time *t* provided that it is open at time 0, which is implemented by the initial condition *n*_0_ = 1


$$n\left(t|0\right)=n_{\infty }+\left(1-n_{\infty }\right)e^{-t/\tau_{n}}$$

The potassium ion channel having four independent identical subunits, this provides the probability that the channel is open at time *t*


$$p\left(t\right)=n^{4}\left(t\right)$$

At steady state


$$p_{\infty }=n_{\infty }^{4}$$

The conditional probability that the channel is open at time *t* provided that the channel is open at time *t* = 0 is given by


p(t∣0)=n4(t∣0)               =[n∞+(1-n∞)e-t/τn]4               =∑q=04(4q)n∞4-q(1-n∞)qe-qt/τn

The autocovariance does follow


CIK(t)=NKiK2C(t)             =NKiK2[p∞p(t∣0)-p∞2]             =NKiK2[n∞4p(t∣0)-(n∞4)2]             =NKiK2n∞4[p(t∣0)-n∞4]             =NKiK2n∞4∑q=14(4q)n∞4-q(1-n∞)qe-qt/τn

where the term corresponding to *q* = 0 in the sum has been canceled with $-n_{\infty }^{4}$.

The power spectrum *S*_*I*_K__ is computed by integrating each term of the sum in *C*_*I*_K__


Sq(ω)=∫0+∞(4q)n∞4-q(1-n∞)qe-qt/τne-iωtdt             =(4q)n∞4-q(1-n∞)q∫0+∞e-qt/τne-iωtdt

Using the non-unitary Fourier transform $F\left[e^{-a|t|}\right]=\frac{2a}{a^{2}+\omega^{2}}$ with angular frequencies and divided by 2 because of the positive half of the time [0; + ∞]


Sq(ω)=(4q)n∞4-q(1-n∞)qq/τnq2/τn2+ω2            =(4q)n∞4-q(1-n∞)qqτnq2+ω2τn2

Applying *S*_*q*_ to each *q*


$$\{\begin{array}{l} S_{1}\left(\omega \right)=4n_{\infty }^{3}\left(1-n_{\infty }\right)\frac{\tau_{n}}{1+\omega^{2}\tau_{n}^{2}}\\ S_{2}\left(\omega \right)=6n_{\infty }^{2}{\left(1-n_{\infty }\right)}^{2}\frac{2\tau_{n}}{4+\omega^{2}\tau_{n}^{2}}\\ S_{3}\left(\omega \right)=4n_{\infty }{\left(1-n_{\infty }\right)}^{3}\frac{3\tau_{n}}{9+\omega^{2}\tau_{n}^{2}}\\ S_{4}\left(\omega \right)={\left(1-n_{\infty }\right)}^{4}\frac{4\tau_{n}}{16+\omega^{2}\tau_{n}^{2}} \end{array}$$

the power spectrum is obtained


(6) $$\begin{array}{l} S_{I_{\text{K}}}\left(\omega \right)=4N_{\text{K}}i_{\text{K}}^{2}n_{\infty }^{4}\left[S_{1}\left(\omega \right)+S_{2}\left(\omega \right)+S_{3}\left(\omega \right)+S_{4}\left(\omega \right)\right] \end{array}$$

There are four Lorentzians with corner frequencies $\omega_{1}=\frac{1}{\tau_{n}}$, $\omega_{2}=\frac{2}{\tau_{n}}$, $\omega_{3}=\frac{3}{\tau_{n}}$, $\omega_{4}=\frac{4}{\tau_{n}}$ respectively. In particular, *jω*_*q*_ correspond to the poles.

The parameters of Table 1 were reused with the potassium channel density $\rho_{\text{K}}=18/\text{μ}{\text{m}}^{2}$, the membrane area $A_{\text{K}}=500\text{μ}{\text{m}}^{2}$, the number of potassium channels *N*_K_ = ρ_K_*A*_K_ and the potassium single-channel conductance γ_K_ = *g*_K_/*N*_K_.

**Table 1:** Parameters of the simulation of the Hodgkin–Huxley equations used to compute impedances given by Equation 2 at different membrane potential displacements *V*_0_.

***C*_*m*_**	**1 μF/cm^2^**
*g*_L_	0.3 mS/cm^2^
*V*_L_	10.6 mV
*g*_K_	36 mS/cm^2^
*V*_K_	–12 mV
*g*_Na_	120 mS/cm^2^
*V*_Na_	120 mV
α_*n*_(*V*)	0.01·(10 − *V*)/(*e*^(10.001 − *V*)/10^ − 1)
β_*n*_(*V*)	0.125·*e*^−*V*/80^
α_*m*_(*V*)	0.1·(25 − *V*)/(*e*^(25 − *V*)/10^ − 1)
β_*m*_(*V*)	4·*e*^−*V*/18^
α_*h*_(*V*)	0.07·*e*^−*V*/20^
β_*h*_(*V*)	1/(*e*^(30 − *V*)/10^ + 1)
Stimulus amplitude	0.0125 mV for Hodgkin–Huxley model, default 0.25 mV for *n*^4^ and *p*_2_ models
Stimulus frequencies	0.2, 0.7, 2, 3, 10, 21, 35, 50, 76, 104, 134, 143, 223, 239, 285, 388, 405, 515, 564, 636, 815, 892, 982 (units in Hz)
Stimulus phases	Pseudo-random values [0;π] rad

**Figure 2:**
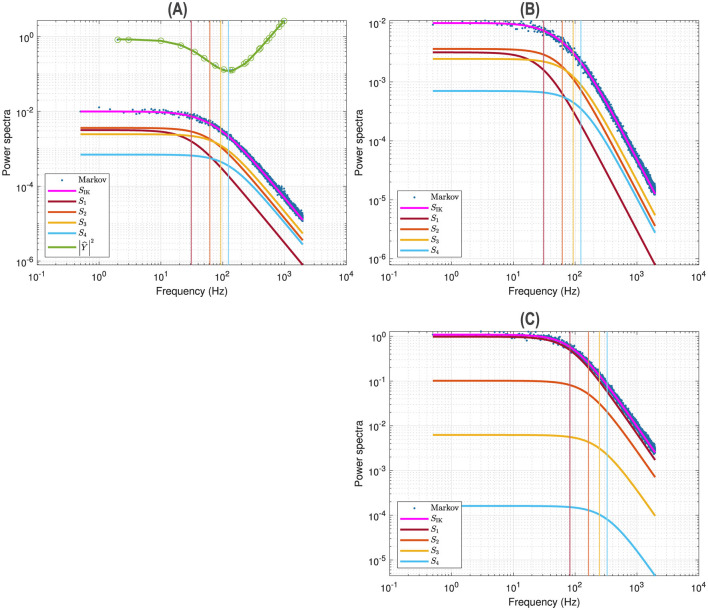
Markov simulations of the *n*^4^ model. The power spectra approximated by 128 iterations are represented by the blue scatterplot. The predicted power spectrum *S*_IK_ (magenta curve) accurately fits the blue scatterplot. The four Lorentzians compose the predicted power spectrum with *S*_1_ (brown curve), *S*_2_ (orange curve), *S*_3_ (yellow curve), *S*_4_ (blue curve). The corresponding corner frequencies ω_1_, ω_2_, ω_3_, ω_4_ are represented by vertical lines. **(A)** Simulations for a 5 mV depolarization with QSA multi-sinusoidal stimulus of amplitude 0.25 mV and frequencies (2, 3, 10, 21, 35, 50, 76, 104, 134, 143, 223, 239, 285, 388, 405, 515, 564, 636, 815, 892, 982) Hertz. The squared admittance |Ŷ|^2^ (green curve) matches the linear Markov responses after adjustment of the vertical offset. **(B)** Simulations for a 5 mV depolarization without stimulus. The fluctuation power spectra are the same as those in **(A)** but there is no admittance. **(C)** Simulations for a 55 mV depolarization without stimulus.

Figure 2A illustrates a voltage clamp Markov simulation (Equation 5) for a 5 mV depolarization of the spontaneous potassium current fluctuations superimposed on the theoretically predicted power spectrum *S*_IK_, the four Lorentzians, the four corner frequencies and the admittance. The simulation was done with the Markov model and QSA multi-sinusoidal stimuli of amplitude 0.25 mV. At this potential, the predicted power spectrum *S*_IK_ (magenta curve) have relative equivalent contributions from all the Lorentzian functions *S*_1_, *S*_2_, *S*_3_, *S*_4_ and certainly not just those present in the lowest corner frequency, $\omega_{4}=\frac{1}{\tau_{n}}$. The voltage responses at the stimulus frequencies are accurately described by the squared admittance |Ŷ|^2^ (green curve) after adjustment of the vertical offset. The square of the admittance is obtained with an input of constant amplitude, which makes it possible to compare it to other curves independent of the input. Figure 2B shows that simulation done without stimulus gave identical power spectra.

### 2.7 Validity

Although this article focuses on potassium channels, the methods are compatible with the full Hodgkin–Huxley model, including sodium channels.

Linear analysis allows the analytical linearization of conductance-based models around a steady state (Mauro, 1970) and their frequency-domain representation using the Fourier transform. This method efficiently captures the behavior of the system under small perturbations.

Quadratic sinusoidal analysis (QSA) investigates nonlinear responses in the frequency domain around a steady state. It supports multi-sinusoidal measurements in both conductance-based models and experimental data (Magnani and Moore, 2011). This method has also been applied beyond Hodgkin–Huxley, including neurons of the prepositus hypoglossi nucleus (Magnani et al., 2013) and neurons of the medial entorhinal cortex (Magnani et al., 2014).

Multi-sinusoidal power spectra extend QSA by averaging multiple measurements while preserving its general applicability. Although demonstrated here for potassium channels, this method is also applicable to other channels, such as sodium.

Stochastic Markov simulations are here considered for potassium channels to focus on the interplay between spontaneous fluctuations and nonlinear responses in a single ion channel type. However, this Markov modeling method is also well-documented for sodium channels (Destexhe and Rudolph-Lilith, 2012; Goldwyn et al., 2011; Goldwyn and Shea-Brown, 2011; O'Donnell and van Rossum, 2014) and continues to evolve in the literature (e.g., Ramlow and Lindner, 2024).

Markov power spectra, computed here for potassium channels, are a method that can be adapted to sodium channels by replacing *n*^4^ with *m*^3^*h*, as described by O'Donnell and van Rossum (2014).

By focusing on potassium channels, this article isolates specific dynamics. Extending to sodium channels in future work would provide complementary insights into neuronal behavior, particularly in understanding the integrated roles of multiple ion channel types.

## 3 Results

### 3.1 The *n*^2^ model

Since chemical relaxation models generally obey the fluctuation-dissipation theorem (FDT), it is useful to compare their linear and nonlinear behaviors using both averaged quadratic (QSA) and noise (Markov) power spectra. This will be done with the potassium conductance of Hodgkin–Huxley equations, namely *n*^4^, and compared with more general relaxation models having fewer sequential states. A similar comparison could be done with the sodium conductance.

A variant of the Hodgkin–Huxley equations, the Frankenhaeuser and Huxley (1964) equations for myelinated nerve, uses a *n*^2^ model for the potassium conductance, which can be simulated as a three-state Markov chain


$$0\overset{2\alpha_{n}}{\underset{\beta_{n}}{⇌}}1\overset{\alpha_{n}}{\underset{2\beta_{n}}{⇌}}2$$

Thus, the *n*^2^ model has only two independent rate constants, α_*n*_ and β_*n*_. More generally, such a *n*^2^ model is a particular sequential kinetic model 0⇌1⇌2, arbitrarily called *p*_2_ in this paper, having the four specific rate constants above with only two independent rate constants. However, a general *p*_2_ model, which has four independent rate constants, will be shown to be a reasonable model for a potassium conductance. In particular, the rate constants of the *p*_2_ model can be selected to show similar behavior to the Hodgkin–Huxley *n*^4^ model. In this case, the *p*_2_ model is a model without exponentiation, which is not identical to the *n*^2^ model, but approximates the Hodgkin–Huxley *n*^4^ model.

### 3.2 The *p*_2_ model

The general *p*_2_ model has four independent rate constants, two forward *k*_1_, *k*_3_, and two backward *k*_4_, *k*_2_, as follows:


(7) $$\begin{array}{l} p_{0}\;\overset{k_{1}}{\underset{k_{2}}{⇌}}\;p_{1}\;\overset{k_{3}}{\underset{k_{4}}{⇌}}\;p_{2} \end{array}$$

The *n*^2^ model is a special case of the *p*_2_ model with *k*_1_ = 2α_*n*_, *k*_3_ = α_*n*_, *k*_4_ = 2β_*n*_ and *k*_2_ = β_*n*_.

More generally, it may be convenient to rewrite *k*_1_, *k*_2_, *k*_3_, *k*_4_ in terms of α_*n*_ and β_*n*_ such that *k*_1_ = *Aα*_*n*_, *k*_3_ = α_*n*_, *k*_4_ = *Bβ*_*n*_ and *k*_2_ = β_*n*_, where *A* and *B* are arbitrary factors. In this way, the *n*^2^ model is a special case of the *p*_2_ model with *A* = *B* = 2.

In this article, the *p*_2_ model simulations use *A* = 0.35 and *B* = 4. The other parameters are the same as the *n*^4^ model simulations given in Table 1.

The master equation is deduced from the three-state Markov chain as follows


(8) $$\begin{array}{l} \{\begin{array}{l} \frac{dp_{0}}{dt}=-k_{1}p_{0}+k_{2}p_{1}\\ \frac{dp_{1}}{dt}=k_{1}p_{0}-\left(k_{2}+k_{3}\right)p_{1}+k_{4}p_{2}\\ \frac{dp_{2}}{dt}=k_{3}p_{1}-k_{4}p_{2} \end{array} \end{array}$$

Since *p*_0_ + *p*_1_ + *p*_2_ = 1, the system can be reduced to the variables *p*_1_ and *p*_2_


(9) $$\begin{array}{l} \{\begin{array}{l} \frac{dp_{1}}{dt}=-\left(k_{1}+k_{2}+k_{3}\right)p_{1}+\left(-k_{1}+k_{4}\right)p_{2}+k_{1}\\ \frac{dp_{2}}{dt}=k_{3}p_{1}-k_{4}p_{2} \end{array} \end{array}$$

This can be rewritten in matrix form


(10) $$\begin{array}{l} \frac{d}{dt}\left(\begin{array}{l} p_{1}\\ p_{2} \end{array}\right)=\left(\begin{array}{ll} \alpha_{11} & \alpha_{12}\\ \alpha_{21} & \alpha_{22} \end{array}\right)\left(\begin{array}{l} p_{1}\\ p_{2} \end{array}\right)+\left(\begin{array}{c} -\alpha_{22}-\alpha_{12}\\ 0 \end{array}\right) \end{array}$$

where


$$\{\begin{array}{l} \alpha_{11}=-\left(k_{1}+k_{2}+k_{3}\right)\\ \alpha_{12}=-k_{1}+k_{4}\\ \alpha_{21}=k_{3}\\ \alpha_{22}=-k_{4} \end{array}$$

At steady state


(11) $$\begin{array}{l} \left(\begin{array}{c} p_{1}^{\infty }\\ p_{2}^{\infty } \end{array}\right)={\left(\begin{array}{cc} \alpha_{11} & \alpha_{12}\\ \alpha_{21} & \alpha_{22} \end{array}\right)}^{-1}\left(\begin{array}{c} \alpha_{22}+\alpha_{12}\\ 0 \end{array}\right)\\ \;=\frac{1}{\alpha_{11}\alpha_{22}-\alpha_{12}\alpha_{21}}\left(\begin{array}{cc} \alpha_{22} & -\alpha_{12}\\ -\alpha_{21} & \alpha_{11} \end{array}\right)\left(\begin{array}{c} \alpha_{22}+\alpha_{12}\\ 0 \end{array}\right) \end{array}$$

(12) $$\begin{array}{l} p_{1}^{\infty }=\frac{\alpha_{22}\left(\alpha_{22}+\alpha_{12}\right)}{\alpha_{11}\alpha_{22}-\alpha_{12}\alpha_{21}},\;p_{2}^{\infty }=\frac{-\alpha_{21}\left(\alpha_{22}+\alpha_{12}\right)}{\alpha_{11}\alpha_{22}-\alpha_{12}\alpha_{21}} \end{array}$$

### 3.3 Linear analysis of the *p*_2_ model

The linear admittance of the *p*_2_ model can be obtained using a method similar to that previously described for the Hodgkin–Huxley model by Mauro (1970). The matrix form given in Equation 10 represents the *p*_2_ model of Equation 9 with *k*_1_, *k*_2_, *k*_3_, *k*_4_ replaced by α_11_, α_12_, α_21_, α_22_ as follows


(13) $$\begin{array}{l} \{\begin{array}{l} \frac{dp_{1}}{dt}=\alpha_{11}p_{1}+\alpha_{12}p_{2}-\left(\alpha_{22}+\alpha_{12}\right),\\ \frac{dp_{2}}{dt}=\alpha_{21}p_{1}+\alpha_{22}p_{2} \end{array} \end{array}$$

The linearization for a small perturbation at steady state is given by


$$\{\begin{array}{l} \delta \frac{dp_{1}}{dt}=\frac{d\alpha_{11}}{dV}\delta Vp_{1}^{\infty }+\alpha_{11}\delta p_{1}+\frac{d\alpha_{12}}{dV}\delta Vp_{2}^{\infty }\\ \;+\alpha_{12}\delta p_{2}-\frac{d\left(\alpha_{22}+\alpha_{12}\right)}{dV}\delta V\\ \delta \frac{dp_{2}}{dt}=\frac{d\alpha_{21}}{dV}\delta Vp_{1}^{\infty }+\alpha_{21}\delta p_{1}+\frac{d\alpha_{22}}{dV}\delta Vp_{2}^{\infty }+\alpha_{22}\delta p_{2} \end{array}$$

or equivalently


$$\{\begin{array}{l} \delta \frac{dp_{1}}{dt}-\alpha_{11}\delta p_{1}-\alpha_{12}\delta p_{2}=[\frac{d\alpha_{11}}{dV}p_{1}^{\infty }+\frac{d\alpha_{12}}{dV}p_{2}^{\infty }\\ \;-\frac{d\left(\alpha_{22}+\alpha_{12}\right)}{dV}]\delta V\\ \delta \frac{dp_{2}}{dt}-\alpha_{21}\delta p_{1}-\alpha_{22}\delta p_{2}=\left[\frac{d\alpha_{21}}{dV}p_{1}^{\infty }+\frac{d\alpha_{22}}{dV}p_{2}^{\infty }\right]\delta V \end{array}$$

Applying the Fourier transform with angular frequency ω


$$\{\begin{array}{l} \left(i\omega -\alpha_{11}\right)\hat{\delta p_{1}}-\alpha_{12}\hat{\delta p_{2}}=[\frac{d\alpha_{11}}{dV}p_{1}^{\infty }+\frac{d\alpha_{12}}{dV}p_{2}^{\infty }\\ \;-\frac{d\left(\alpha_{22}+\alpha_{12}\right)}{dV}]\hat{\delta V}\\ \left(i\omega -\alpha_{22}\right)\hat{\delta p_{2}}-\alpha_{21}\hat{\delta p_{1}}=\left[\frac{d\alpha_{21}}{dV}p_{1}^{\infty }+\frac{d\alpha_{22}}{dV}p_{2}^{\infty }\right]\hat{\delta V} \end{array}$$

Then $\hat{\delta p_{2}}$ can be deduced from


$$\{\begin{array}{l} \;\alpha_{21}\left(i\omega -\alpha_{11}\right)\hat{\delta p_{1}}-\alpha_{21}\alpha_{12}\hat{\delta p_{2}}=\alpha_{21}\left[\frac{d\alpha_{11}}{dV}p_{1}^{\infty }+\frac{d\alpha_{12}}{dV}p_{2}^{\infty }-\frac{d\left(\alpha_{22}+\alpha_{12}\right)}{dV}\right]\hat{\delta V}\\ \left(i\omega -\alpha_{11}\right)\left(i\omega -\alpha_{22}\right)\hat{\delta p_{2}}-\left(i\omega -\alpha_{11}\right)\alpha_{21}\hat{\delta p_{1}}=\left(i\omega -\alpha_{11}\right)\left[\frac{d\alpha_{21}}{dV}p_{1}^{\infty }+\frac{d\alpha_{22}}{dV}p_{2}^{\infty }\right]\hat{\delta V} \end{array}$$

By summation


$$\begin{array}{l} \begin{array}{l} \left[\left(i\omega -\alpha_{11}\right)\left(i\omega -\alpha_{22}\right)-\alpha_{21}\alpha_{12}\right]\hat{\delta p_{2}}\\ =\alpha_{21}\left[\frac{d\alpha_{11}}{dV}p_{1}^{\infty }+\frac{d\alpha_{12}}{dV}p_{2}^{\infty }-\frac{d\left(\alpha_{22}+\alpha_{12}\right)}{dV}\right]\hat{\delta V}\\ +\left(i\omega -\alpha_{11}\right)\left[\frac{d\alpha_{21}}{dV}p_{1}^{\infty }+\frac{d\alpha_{22}}{dV}p_{2}^{\infty }\right]\hat{\delta V} \end{array} \end{array}$$

This implies the rate of variation


$$\begin{array}{l} \frac{\hat{\delta p_{2}}}{\hat{\delta V}}=\frac{\begin{array}{l} \alpha_{21}\left(\frac{d\alpha_{11}}{dV}p_{1}^{\infty }+\frac{d\alpha_{12}}{dV}p_{2}^{\infty }-\frac{d\left(\alpha_{22}+\alpha_{12}\right)}{dV}\right)\\ +\left(i\omega -\alpha_{11}\right)\left(\frac{d\alpha_{21}}{dV}p_{1}^{\infty }+\frac{d\alpha_{22}}{dV}p_{2}^{\infty }\right) \end{array}}{\left(i\omega -\alpha_{11}\right)\left(i\omega -\alpha_{22}\right)-\alpha_{21}\alpha_{12}} \end{array}$$

The current across the lipid bilayer is given by


$$I=C_{m}\frac{dV}{dt}+g_{\text{L}}\left(V-V_{\text{L}}\right)+g_{\text{K}}p_{2}\left(V-V_{\text{K}}\right)$$

It is linearized by


$$\delta I=C_{m}\frac{d\left(\delta V\right)}{dt}+g_{\text{L}}\delta V+g_{\text{K}}\left[\delta p_{2}\left(V_{0}-V_{\text{K}}\right)+p_{2}^{\infty }\delta V\right]$$

Applying the Fourier transform with angular frequency ω


$$\hat{\delta I}=C_{m}i\omega \hat{\delta V}+g_{\text{L}}\hat{\delta V}+g_{\text{K}}\left[\hat{\delta p_{2}}\left(V_{0}-V_{\text{K}}\right)+p_{2}^{\infty }\hat{\delta V}\right]$$

This provides the admittance of the *p*_2_ model


$$\hat{Y}=\frac{\hat{\delta I}}{\hat{\delta V}}=C_{m}i\omega +g_{\text{L}}+g_{\text{K}}\left(\frac{\hat{\delta p_{2}}}{\hat{\delta V}}\left(V_{0}-V_{\text{K}}\right)+p_{2}^{\infty }\right)$$

### 3.4 Markov power spectra of the *p*_2_ model

From Equations 10, 11, the differential equation can be written in the homogeneous form


$$\frac{d}{dt}\left(\begin{array}{l} p_{1}-p_{1}^{\infty }\\ p_{2}-p_{2}^{\infty } \end{array}\right)=\left(\begin{array}{ll} \alpha_{11} & \alpha_{12}\\ \alpha_{21} & \alpha_{22} \end{array}\right)\left(\begin{array}{l} p_{1}-p_{1}^{\infty }\\ p_{2}-p_{2}^{\infty } \end{array}\right)$$

More concisely


$$\frac{d\text{p}}{dt}=\text{M}\text{p}$$

where $\text{p}=\left(\begin{array}{c} p_{1}-p_{1}^{\infty }\\ p_{2}-p_{2}^{\infty } \end{array}\right)$ and $\text{M}=\left(\begin{array}{cc} \alpha_{11} & \alpha_{12}\\ \alpha_{21} & \alpha_{22} \end{array}\right)$.

The general solution is a linear combination


(14) $$\begin{array}{l} \text{p}=c_{1}e^{{\text{λ}}_{1}t}{\text{v}}_{1}+c_{2}e^{{\text{λ}}_{2}t}{\text{v}}_{2} \end{array}$$

where λ_1_, λ_2_ are eigenvalues of **M**, **v_1_**, **v_2_** are eigenvectors of **M** and *c*_1_, *c*_2_ are constants. Indeed


$$\begin{array}{l} \text{M}\text{p}=c_{1}e^{{\text{λ}}_{1}t}\text{M}{\text{v}}_{1}+c_{2}e^{{\text{λ}}_{2}t}\text{M}{\text{v}}_{2}\\ \;=c_{1}e^{{\text{λ}}_{1}t}{\text{λ}}_{1}{\text{v}}_{1}+c_{2}e^{{\text{λ}}_{2}t}{\text{λ}}_{2}{\text{v}}_{2}\\ \;=\frac{d\text{p}}{dt} \end{array}$$

The trace *T* = α_11_ + α_22_ and determinant *D* = α_11_α_22_ − α_12_α_21_ of **M** determine the eigenvalues


$$\begin{array}{c} {\text{λ}}_{1,2}=\frac{T\pm \sqrt{T^{2}-4D}}{2}\\ {\text{λ}}_{1,2}=\frac{\alpha_{11}+\alpha_{22}\pm \sqrt{{\left(\alpha_{11}+\alpha_{22}\right)}^{2}-4\left(\alpha_{11}\alpha_{22}-\alpha_{12}\alpha_{21}\right)}}{2} \end{array}$$

Since *p* represents probabilities, λ_1_ and λ_2_ must be negative to have exponential decreases rather than exponential increases. This determines two time constants for the *p*_2_ model


$$\tau_{1}=-\frac{1}{{\text{λ}}_{1}},\;\tau_{2}=-\frac{1}{{\text{λ}}_{2}}$$

The eigenvectors **v_1_** and **v_2_** are obtained by solving the equation **Mv** = λ**v** and substituting eigenvalues λ = λ_1_ and λ = λ_2_


$${\text{v}}_{1}=\left(\begin{array}{c} \frac{{\text{λ}}_{1}-\alpha_{22}}{\alpha_{21}}\\ 1 \end{array}\right),\;{\text{v}}_{2}=\left(\begin{array}{c} \frac{{\text{λ}}_{2}-\alpha_{22}}{\alpha_{21}}\\ 1 \end{array}\right)$$

Inserting **v_1_** and **v_2_** into Equation 14


$$\begin{array}{c} \left(\begin{array}{c} p_{1}-p_{1}^{\infty }\\ p_{2}-p_{2}^{\infty } \end{array}\right)=c_{1}e^{{\text{λ}}_{1}t}\left(\begin{array}{c} \frac{{\text{λ}}_{1}-\alpha_{22}}{\alpha_{21}}\\ 1 \end{array}\right)+c_{2}e^{{\text{λ}}_{2}t}\left(\begin{array}{c} \frac{{\text{λ}}_{2}-\alpha_{22}}{\alpha_{21}}\\ 1 \end{array}\right) \end{array}$$

The time *t* = 0 determines the coefficients *c*_1_ and *c*_2_ as follows


$$\begin{array}{c} \{\begin{array}{l} p_{1}\left(0\right)-p_{1}^{\infty }=c_{1}\frac{{\text{λ}}_{1}-\alpha_{22}}{\alpha_{21}}+c_{2}\frac{{\text{λ}}_{2}-\alpha_{22}}{\alpha_{21}}\\ p_{2}\left(0\right)-p_{2}^{\infty }=c_{1}+c_{2} \end{array}\\ \{\begin{array}{l} p_{1}\left(0\right)-p_{1}^{\infty }=c_{1}\frac{{\text{λ}}_{1}-\alpha_{22}}{\alpha_{21}}+\left(p_{2}\left(0\right)-p_{2}^{\infty }-c_{1}\right)\frac{{\text{λ}}_{2}-\alpha_{22}}{\alpha_{21}}\\ \;c_{2}=p_{2}\left(0\right)-p_{2}^{\infty }-c_{1} \end{array}\\ \{\begin{array}{l} c_{1}\left(\frac{{\text{λ}}_{2}-{\text{λ}}_{1}}{\alpha_{21}}\right)=p_{2}\left(0\right)\frac{{\text{λ}}_{2}-\alpha_{22}}{\alpha_{21}}-p_{2}^{\infty }\frac{{\text{λ}}_{2}-\alpha_{22}}{\alpha_{21}}-p_{1}\left(0\right)+p_{1}^{\infty }\\ \;c_{2}=p_{2}\left(0\right)-p_{2}^{\infty }-c_{1} \end{array}\\ \{\begin{array}{l} c_{1}=\frac{\left({\text{λ}}_{2}-\alpha_{22}\right)\left(p_{2}\left(0\right)-p_{2}^{\infty }\right)-\alpha_{21}\left(p_{1}\left(0\right)-p_{1}^{\infty }\right)}{{\text{λ}}_{2}-{\text{λ}}_{1}}\\ c_{2}=p_{2}\left(0\right)-p_{2}^{\infty }-c_{1} \end{array} \end{array}$$

The power spectrum can be calculated using the same reasoning as with the model *n*^4^ previously because it is a sequential model with three states instead of five states. In particular, the single-channel autocovariance is based on the conditional probability *p*_2_(*t*∣0) and the steady state probability $p_{2}^{\infty }$


$$C\left(t\right)=p_{2}^{\infty }\cdot p_{2}\left(t|0\right)-{\left(p_{2}^{\infty }\right)}^{2}$$

Considering the unique state (*p*_0_, *p*_1_, *p*_2_) = (0, 0, 1) when the channel is open


$$\{\begin{array}{l} c_{1}=\frac{\left({\text{λ}}_{2}-\alpha_{22}\right)\left(1-p_{2}^{\infty }\right)+\alpha_{21}p_{1}^{\infty }}{{\text{λ}}_{2}-{\text{λ}}_{1}}\\ c_{2}=1-p_{2}^{\infty }-c_{1} \end{array}$$

As a result, the conditional probability *p*_2_(*t*|0) is given by


$$p_{2}\left(t|0\right)=p_{2}^{\infty }+c_{1}e^{{\text{λ}}_{1}t}+c_{2}e^{{\text{λ}}_{2}t}$$

The autocovariance is given by


$$\begin{array}{l} C\left(t\right)=p_{2}^{\infty }\left[p_{2}^{\infty }+c_{1}e^{{\text{λ}}_{1}t}+c_{2}e^{{\text{λ}}_{2}t}\right]-{\left(p_{2}^{\infty }\right)}^{2}\\ \;=p_{2}^{\infty }c_{1}e^{{\text{λ}}_{1}t}+p_{2}^{\infty }c_{2}e^{{\text{λ}}_{2}t} \end{array}$$

This implies autocovariance *C*_*I*_K__(*t*) of the total fluctuating current


$$\begin{array}{l} C_{I_{\text{K}}}\left(t\right)=N_{\text{K}}i_{\text{K}}^{2}C\left(t\right)\\ \;=N_{\text{K}}i_{\text{K}}^{2}p_{2}^{\infty }\left(c_{1}e^{{\text{λ}}_{1}t}+c_{2}e^{{\text{λ}}_{2}t}\right) \end{array}$$

Following the same conventions as previously, the power spectrum is


$$\begin{array}{l} S_{I_{\text{K}}}\left(\omega \right)=2S_{I_{\text{K}}}^{\pm }\left(\omega \right)\\ \;=4ℜ\int_{0}^{+\infty }C_{I_{\text{K}}}\left(t\right)e^{-i\omega t}dt\\ \;=4N_{\text{K}}i_{\text{K}}^{2}p_{2}^{\infty }ℜ\int_{0}^{+\infty }\left(c_{1}e^{{\text{λ}}_{1}t}+c_{2}e^{{\text{λ}}_{2}t}\right)e^{-i\omega t}dt \end{array}$$

There are two integrals to be calculated for each number *q* = 1, 2


$$S_{q}\left(\omega \right)=\int_{0}^{+\infty }c_{q}e^{{\text{λ}}_{q}t}e^{-i\omega t}dt$$

Using the non-unitary Fourier transform $F\left[e^{-a|t|}\right]=\frac{2a}{a^{2}+\omega^{2}}$ with angular frequencies and divided by 2 because of the positive half of the time [0; + ∞]


$$S_{q}\left(\omega \right)=c_{q}\frac{-{\text{λ}}_{q}}{{\text{λ}}_{q}^{2}+\omega^{2}}$$

Then, the power spectrum is deduced


$$S_{I_{\text{K}}}\left(\omega \right)=4N_{\text{K}}i_{\text{K}}^{2}p_{2}^{\infty }\left(c_{1}\frac{-{\text{λ}}_{1}}{{\text{λ}}_{1}^{2}+\omega^{2}}+c_{2}\frac{-{\text{λ}}_{2}}{{\text{λ}}_{2}^{2}+\omega^{2}}\right)$$

By using the time constants $\tau_{1}=-\frac{1}{\lambda_{1}}$ and $\tau_{2}=-\frac{1}{\lambda_{2}}$


$$\begin{array}{l} S_{I_{\text{K}}}\left(\omega \right)=4N_{\text{K}}i_{\text{K}}^{2}p_{2}^{\infty }\left(c_{1}\frac{1/\tau_{1}}{1/\tau_{1}^{2}+\omega^{2}}+c_{2}\frac{1/\tau_{2}}{1/\tau_{2}^{2}+\omega^{2}}\right)\\ \;=4N_{\text{K}}i_{\text{K}}^{2}p_{2}^{\infty }\left(c_{1}\frac{\tau_{1}}{1+\omega^{2}\tau_{1}^{2}}+c_{2}\frac{\tau_{2}}{1+\omega^{2}\tau_{2}^{2}}\right) \end{array}$$

**Figure 3:**
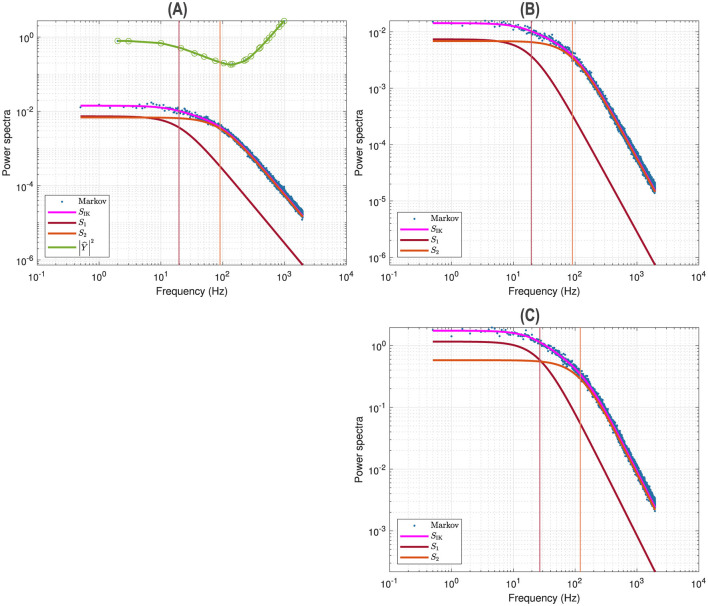
Markov simulations of the *p*_2_ model. The power spectra approximated by 128 iterations are represented by the blue scatterplot. The predicted power spectrum *S*_*I*_K__ (magenta curve) accurately fits the blue scatterplot. The two Lorentzians compose the predicted power spectrum with *S*_1_ (brown curve) and *S*_2_ (orange curve). The corresponding corner frequencies ω_1_ and ω_2_ are represented by vertical lines. **(A)** Simulations for a 5 mV depolarization with QSA multi-sinusoidal stimulus of amplitude 0.25 mV and frequencies (2, 3, 10, 21, 35, 50, 76, 104, 134, 143, 223, 239, 285, 388, 405, 515, 564, 636, 815, 892, 982) Hertz. The squared admittance |Ŷ|^2^ (green curve) matches the linear Markov responses after adjustment of the vertical offset. **(B)** Simulations for a 5 mV depolarization without stimulus. The fluctuation power spectra are the same as those in **(A)** but there is no admittance. **(C)** Simulations for a 55 mV depolarization without stimulus. The rate constants have been manually selected to approximate the frequency domain responses of the *n*^4^ model, which can be compared to Figure 2C.

Figure 3A illustrates a voltage clamp Markov simulation for a 5 mV depolarization of the spontaneous potassium current fluctuations superimposed on the theoretically predicted power spectrum *S*_IK_, the two Lorentzians, the two corner frequencies and the squared admittance |Ŷ|^2^. The simulation was done with the Markov model and QSA multi-sinusoidal stimuli of amplitude 0.25 mV. At this potential, the predicted power spectrum *S*_IK_ (magenta curve) is well described by a single Lorentzian function for frequencies greater than the corner frequency. The square of the admittance is obtained with an input of constant amplitude, which makes it possible to compare it to other curves independent of the input. Figure 3B shows that simulations done without stimulus gave identical power spectra.

### 3.5 Neuronal functions compared to neuronal fluctuations

Individual neurons are characterized both by deterministic neuronal functions, such as those revealed by linear and quadratic analysis (QSA), and by random fluctuations, such as those produced by Markov models. Both behaviors play an essential role in subthreshold neuronal processing to generate action potentials and, consequently, for neuronal networks in general.

Both behaviors are fundamentally related to the nonlinear kinetic processes underlying ion channels, such as the *n*^4^ model, its *n*^2^ simplification, or the generalization of *n*^2^ to the *p*_2_ model without exponentiation, as described above. In contrast, the fluctuation-dissipation theorem implies that ion channel fluctuations should be described by a linearization of the underlying nonlinear channel kinetics. To address these issues, the sequential kinetic model *p*_2_ described above is an approximate alternative to the Hodgkin–Huxley model that may be useful because it is not based on exponentiation. In all cases, Markov models are considered an adequate approach to simulate the fluctuations of a single-channel. Other neuronal noise fluctuation models are of interest, such as stochastic differential equations (SDEs), but are not considered in this paper. Since sequential kinetic models include Hodgkin–Huxley models with exponentiation as a special case, and have been shown to describe kinetic behavior as well as or better than the Hodgkin–Huxley model, it is appropriate to compare the simple *p*_2_ model with the *n*^4^ model. This will be done in the following voltage clamp simulations for the model with nonlinear exponentiation *n*^4^ and the model without exponentiation *p*_2_ to provide a comparison of Markov fluctuations, their linear and quadratic (QSA) stimulated behavior. A fundamental question is whether models with exponentiation of the Hodgkin–Huxley type or models without exponentiation are the best descriptions of a single neuron behavior. These fluctuation simulations determine whether or not the spontaneous current noise at a fixed voltage clamped membrane potential should be based on a nonlinear exponentiation such that *n*^4^.

The control case for nonlinear exponentiation is the Hodgkin–Huxley *n*^4^ model. The frequency domain analysis is performed on simulated data measurements at two different depolarized membrane potentials, namely 5 mV and 55 mV, such as one could record from a voltage clamped biological neuron. These two voltage clamp potentials were chosen to be either near the resting value or full activation of the voltage-dependent ionic conductances. Simulations were performed using MATLAB R2022b (MathWorks) with the nonlinear classical Hodgkin–Huxley *n*^4^ model described in above sections. Two modes have been considered, deterministic and stochastic, based on the programs of Goldwyn et al. (2011) and Goldwyn and Shea-Brown (2011). The deterministic mode was done using Euler's method for ODE, while the stochastic mode was based on Gillespie's algorithm. The results of the numerical simulations are superimposed on an analytical form for the impedance (Equation 2) and the Markov fluctuations (Equation 6). Thus, the simulations are analogous to measured data from a biological neuron, which can be analyzed in the frequency domain using the methods described above. As demonstrated by Magnani et al. (2013), this type of frequency domain analysis is an especially efficient and useful way to determine the accuracy of a particular model, since frequency domain analysis of data from biological neurons is a sensitive experimental measure that can be rigorously compared to model predictions. When data are produced by a model, as in this paper, comparisons between numerical simulations and analytical expressions of impedance and Markov fluctuations are used to check the accuracy of calculations. In addition, the QSA method provides a nonlinear analysis independent of a particular type of model or experiment, and thus can be applied to compare simulated data for different models.

**Figure 4:**
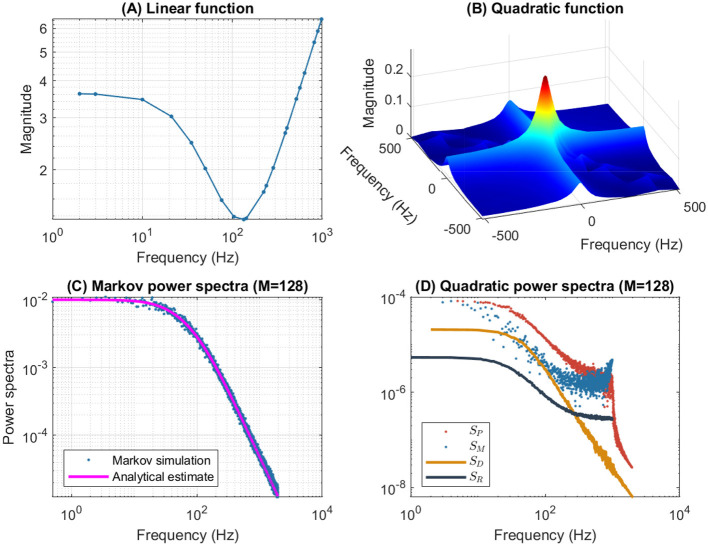
Frequency analysis of the *n*^4^ model for a 5 mV depolarization. **(A)** Linear admittance (smooth line) generated by a QSA multi-sinusoidal stimulus of amplitude 0.25 mV and frequencies (2, 3, 10, 21, 35, 50, 76, 104, 134, 143, 223, 239, 285, 388, 405, 515, 564, 636, 815, 892, 982) Hertz. The linear responses at stimulating frequencies are marked by small circles. **(B)** QSA matrix in 3D representation obtained with the same stimulus as the linear function, but the plot is cut at 500 Hz for a better readability. The color code indicates the amplitude of the quadratic response. Each value (ω_*i*_, ω_*j*_, |*Q*_*i, j*_|) in the 3D plot represents the magnitude of the quadratic response to a frequency interaction. **(C)** Power spectra of the Markov simulation (128 iterations) superimposed on the analytical estimate. The frequencies are continuous up to twice the maximum stimulus frequency to include the highest QSA frequency (frequency doubling). The analytical estimation provides an excellent control of the Markov simulation. **(D)** Quadratic power spectra averaged over several ODE simulations (128 iterations) for different sets of QSA frequencies randomized up to 1,000 Hz. Quadratic responses are represented at their particular frequencies, namely *S*_*P*_(|ω_*i*_| + |ω_*j*_|) at frequency sums (red points), *S*_*M*_(||ω_*i*_| − |ω_*j*_||) at frequency differences (blue points), *S*_*D*_(2|ω_*k*_|) at frequency doubling (orange points) and *S*_*R*_(|ω_*j*_|) for the mean squared quadratic output by matrix columns (black points). Unlike the QSA matrix which is based on ratios of outputs to inputs, quadratic power spectra are evaluated directly from output measurements like Markov power spectra.

Figure 4 illustrates the frequency analysis of the *n*^4^ model for a 5 mV depolarization. The upper left plot (Figure 4A) shows a typical low frequency linear admittance anti-resonance (smooth line) generated by a QSA multi-sinusoidal stimulus of amplitude 0.25 mV. The upper right plot (Figure 4B) shows the QSA matrix as a 3D representation, an intersection of lines on the plane for any two linear frequencies ω_*i*_, ω_*j*_ represents an interactive quadratic frequency ω_*i*_ + ω_*j*_ for *i, j* ∈ {−*N*, …, −1, +1, …, +*N*} and the color code indicates the amplitude of the quadratic response. Remarkably, the quadratic response shows no anti-resonance. The lower left plot (Figure 4C) shows the power spectra of the Markov simulation superimposed on the analytical estimate of the Equation 6. It is clear that the analytical estimation provides an excellent control of the Markov simulation, both of which reveal the characteristics of a low-pass filter. The lower right plot (Figure 4D) shows the quadratic power spectra averaged over several ODE simulations for different random sets of QSA frequencies. Indeed, since the QSA frequency sets have few frequencies, it is necessary to average several measurements to increase the accuracy of the power spectra. Individual amplitudes of the various quadratic responses are represented at their particular frequencies, namely *S*_*P*_(|ω_*i*_| + |ω_*j*_|) at frequency sums (red points), *S*_*M*_(||ω_*i*_| − |ω_*j*_||) at frequency differences (blue points), *S*_*D*_(2|ω_*k*_|) at frequency doubling (orange points) and *S*_*R*_(|ω_*j*_|) for the mean squared quadratic output by matrix columns (black points). All of these responses exhibit low-pass filter characteristics, but they flatten out by reaching a constant value at high frequencies, with the exception of *S*_*D*_(2|ω_*k*_|) at frequency doubling for which the amplitudes decrease at high frequencies. Unlike the QSA matrix which is based on ratios of outputs to inputs, quadratic power spectra are evaluated directly from output measurements like Markov power spectra.

The amplitudes of the quadratic power spectra are smaller than those of the Markov power spectra. Indeed, quadratic responses are an order of magnitude smaller than linear responses, while the fluctuation-dissipation theorem relates spontaneous fluctuations to linear responses. In particular, Markov power spectra are based on autocorrelation, which corresponds to the second order cumulant, whereas higher order spectra would involve at least the third order cumulant as explained by Mendel (1991). At this end of this paper, the discussion provides some simulations to compare the amplitudes of quadratic responses and spontaneous fluctuations.

It is well known that nonlinear systems can generate frequency mixing processes, such as those producing interactive frequencies |ω_*i*_| + |ω_*j*_|, ||ω_*i*_| − |ω_*j*_|| and 2|ω_*k*_|. Frequency mixing behavior is fundamental in some scientific fields, such as nonlinear optics, as described for example by Boyd (2008). Frequency mixing introduces complexity into the response, which may contain frequencies that are not present in the stimulus. Thus, neurons mix stimulus oscillations into quadratic responses that may appear less uniform than linear responses. In particular, the power spectra *S*_*P*_(|ω_*i*_| + |ω_*j*_|) and *S*_*M*_(||ω_*i*_| − |ω_*j*_||) show scatter plots with a lot of dispersion that look like stochastic fluctuations. It should be noted that although different random sets of QSA frequencies were used for averaging, each individual QSA matrix is obtained from a deterministic ODE. Randomizing the frequencies only extends the range of frequencies for analysis, implying that the dispersion is due to the quadratic neuronal function rather than a lack of frequencies. In contrast, frequency doubling generates power spectra *S*_*D*_(2|ω_*k*_|) that approximately follow a smooth line. This is because frequency doubling is calculated along the frequency diagonal (ω_*k*_, ω_*k*_) on the 3D representation, which decreases smoothly from low frequencies (color-coded red for height) to high frequencies (color-coded blue for height). Similarly, the power spectra *S*_*R*_(|ω_*j*_|) for the mean squared quadratic output by matrix columns appear to be quite smooth for the reason that the 3D representation is smooth and summed column by column. Remarkably, the power spectra *S*_*D*_(2|ω_*k*_|) tends to zero at high frequencies due to the attenuation of quadratic responses along the diagonal (ω_*k*_, ω_*k*_), while the power spectra *S*_*R*_(|ω_*j*_|) flatten at high frequencies because of the residual quadratic responses around the horizontal (*x*, 0) and vertical axes (0, *y*). Therefore, the irregularity of the power spectra *S*_*P*_(|ω_*i*_| + |ω_*j*_|) and *S*_*M*_(||ω_*i*_| − |ω_*j*_||) are fundamentally due to non-trivial quadratic frequency interactions between ω_*i*_ and ω_*j*_ when they do not follow the smooth shape of the 3D representation (by diagonal or by columns).

**Figure 5:**
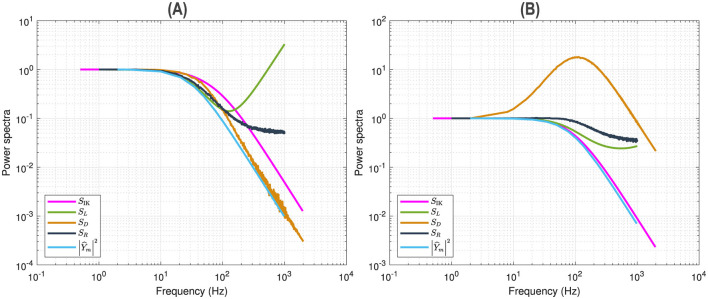
Power spectral analysis of the *n*^4^ model. Comparison between the analytical estimate of Markov fluctuations *S*_IK_ (magenta), the linear power spectrum *S*_*L*_ (green), the quadratic power spectra *S*_*D*_ (orange) and *S*_*R*_ (black), the modified squared admittance $|{Ŷ}_{m}{|}^{2}$ (blue). **(A)** Simulations for a 5 mV depolarization. **(B)** Simulations for a 55 mV depolarization.

Figure 5A shows the superposition of five curves of the *n*^4^ model for a 5 mV depolarization. This allows a comparison of the linear, nonlinear and fluctuation amplitudes behavior. The curves were scaled to unity at low frequencies. The curve *S*_IK_ represents the analytical estimate of Markov fluctuations. The curve *S*_*L*_ represents the linear power spectrum at fundamental frequencies. The curve *S*_*D*_ represents the quadratic power spectrum at frequency doubling. The curve *S*_*R*_ represents the mean squared quadratic output by matrix columns. The multi-sinusoidal power spectra *S*_*L*_, *S*_*D*_, *S*_*R*_ were averaged over different random sets of non-overlapping frequencies as in Figure 4. The curve $|{Ŷ}_{m}{|}^{2}$ represents the squared admittance modified from the Equation 1 without the membrane capacitance nor the frequency independent terms, namely


$${\hat{Y}}_{m}=g_{\text{K}}\left[4n_{0}^{3}\left(V_{0}-V_{\text{K}}\right)\hat{D_{n}}\right]$$

The contribution of the capacitance to the actual admittance is in part responsible for an anti-resonance that leads to high frequency responses that are dominant, thus preventing a direct comparison of the linearized admittance with nonlinear responses or fluctuation behavior. The square of the admittance is obtained with an input of constant amplitude, which makes it possible to compare it to other curves independent of the input.

The fall of the modified squared admittance $|{Ŷ}_{m}{|}^{2}$ is more marked than that of the Markov fluctuations *S*_IK_, which is also the case for the linear power spectrum *S*_*L*_ over a limited range of frequencies before the reversal. The fall of the quadratic power spectra *S*_*D*_ and *S*_*R*_ is more marked than that of the Markov fluctuations *S*_IK_ but less than that of the modified squared admittance, as well as compared to the linear power spectrum *S*_*L*_ before the reversal. As explained previously, *S*_*D*_ and *S*_*R*_ follow the smooth 3D representation of the QSA matrices, in particular *S*_*D*_ decreases indefinitely while *S*_*R*_ flattens at high frequencies.

These results show that the evoked nonlinear responses have a lower frequency behavior than spontaneous fluctuations, suggesting that Markov fluctuations have a different origin than the nonlinearity evoked by stimulus despite nonlinearity of rate constants or exponentiation *n*^4^ in the stochastic equations.

**Figure 6:**
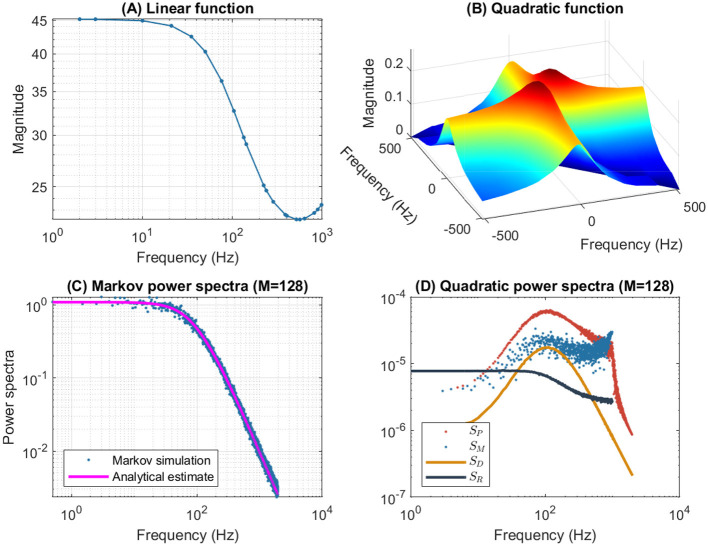
Frequency analysis of the *n*^4^ model for a 55 mV depolarization. The plots were generated using the same presentation as in Figure 4. **(A)** Linear function. **(B)** Quadratic function. **(C)** Markov power spectra (*M* = 128). **(D)** Quadratic power spectra (*M* = 128).

Figure 6 shows the frequency analysis of the *n*^4^ model for a 55 mV depolarization, which is very different from the previous 5 mV depolarization, clearly illustrating the voltage dependence of the potassium channel in both the linear and nonlinear analyses. The upper left plot shows linear admittance (smooth line) generated by a QSA multi-sinusoidal stimulus of amplitude 0.25 mV, which reveals the onset of a high frequency anti-resonance minimum. The upper right plot shows the QSA matrix as a 3D representation, which is increased in a slightly shifted bandwidth from the previous depolarization of 5 mV. The lower left plot shows the power spectra of the Markov simulation superimposed on the analytical estimate, which is similar to a low-pass filter as for a depolarization of 5 mV. The lower right plot shows the quadratic power spectra averaged over several ODE simulations for different random sets of QSA frequencies. The individual components *S*_*P*_, *S*_*M*_, *S*_*D*_ confirms the slightly shifted bandwidth observed on the QSA matrix, revealing more precisely the marked resonance. In particular, the nonlinear resonant frequencies are clearly lower than that of the linear anti-resonance. However, the *S*_*R*_ component based on each matrix column has low-pass filter characteristics. Thus, low-pass behavior is observed at 5 and 55 mV for the Markov simulation of and the column-mean-square *S*_*R*_. Interestingly, the nonlinear behavior for highly activated conductances shows resonance in the quadratic responses at frequencies different from the anti-resonance observed for the linear response.

Figure 5B (55 mV) shows the superposition of five curves of the *n*^4^ model for a 55 mV depolarization, using the same presentation as in Figure 5A (5 mV). The modified squared admittance $|{Ŷ}_{m}{|}^{2}$ nearly superimposes on the Markov fluctuations *S*_IK_, which is an effect of the voltage-dependent potassium conductance induced by the 55 mV depolarization.

Also, by comparing Figure 2C (55 mV) and Figure 2B (5 mV), each of the four relaxation time constants clearly appears as a function of the membrane potential and at large depolarizations the slowest time constant is dominant, i.e. the component *S*_1_ fits the Markov fluctuations *S*_IK_ accurately.

Thus, at 55 mV depolarization, the fluctuation behavior *S*_IK_ is dominated by *S*_1_ and has a kinetic behavior similar to that of a modified squared admittance $|{Ŷ}_{m}{|}^{2}$, which does not occur at the less depolarized potential 5 mV shown above.

The linear power spectrum *S*_*L*_ is similar to the modified squared admittance $|{Ŷ}_{m}{|}^{2}$ at low frequencies, but it does not fall at high frequencies due to the membrane capacitance and frequency independent terms. The column-mean-square component *S*_*R*_ has a higher frequency content than the linear behavior, although less dramatic than the marked scaled resonance of the frequency doubling component *S*_*D*_.

In summary, the power spectra of the QSA responses for the *n*^4^ model are clearly different than those of Markov fluctuations and the power spectra of the linear responses do not generally follow that of the spontaneous fluctuations. Thus, Markov fluctuations are not predicted by small signal evoked linear or quadratic responses. Interestingly, Markov fluctuations at depolarized membrane potentials are dominated by the linear time constant, this is not generally the case for lesser depolarizations, as illustrated between a 5 and 55 mV depolarizations.

### 3.6 Observations on the linear fluctuation-dissipation theorem

As discussed by Stevens (1972), the power *n*^4^ in Hodgkin–Huxley equations leads to two interpretations of the origin of potassium conductance noise. The first case involves Markov simulations and power spectra with four Lorentzian terms, as illustrated in Figure 2B. The second case involves linearization of the Hodgkin–Huxley equations with a single relaxation time, as illustrated by the term $g_{\text{K}}\left[4n_{0}^{3}\left(V_{0}-V_{\text{K}}\right)\hat{D_{n}}+n_{0}^{4}\right]$ in Equation 1.

The linear fluctuation-dissipation theorem states that the spontaneous fluctuations should have the same kinetic behavior as a response to a small signal, namely a linear response. The fact that Markov simulations have four time constants whereas the linearization has a single relaxation time does not agree with the linear fluctuation-dissipation theorem. Thus, the linear fluctuation-dissipation theorem does not hold for the *n*^4^ model. This suggests exploring nonlinear extensions of the linear fluctuation-dissipation theorem in the physics literature. However, it is also interesting to consider the case when Markov simulations have fewer time constants, as with the *p*_2_ model described in this article.

### 3.7 Comparison between *n*^4^ and *p*_2_ models

#### 3.7.1 Depolarization of 55 mV

As discussed above, sequential kinetic models can produce the exponentiation of the *n*^4^ Hodgkin–Huxley model, so that the probability of the channel opening coincides with *n*^4^. Similarly, for the *n*^2^ model, the probability of the channel being open coincides with *n*^2^. However, the *p*_2_ model generalizes the rate constants of the *n*^2^ model, so that the probability of the channel opening is not necessarily an exponentiation. Thus, sequential kinetic models are more general than the Hodgkin–Huxley model, as that they do not require exponentiation. In these models, the nonlinear voltage dependence of neuronal ionic conductances lies essentially in the rate constants between sequential states and not in an exponentiation functional dependence of the conductance gating variable.

If such non-exponentiation-based sequential kinetic models also fit the data, then their nonlinear behavior may more realistically describe the underlying molecular mechanisms of ion channel activity. Indeed, in accordance with the principle of parsimony, these models free themselves from the constraint that the probability of channel opening must be an exponentiation.

**Figure 7:**
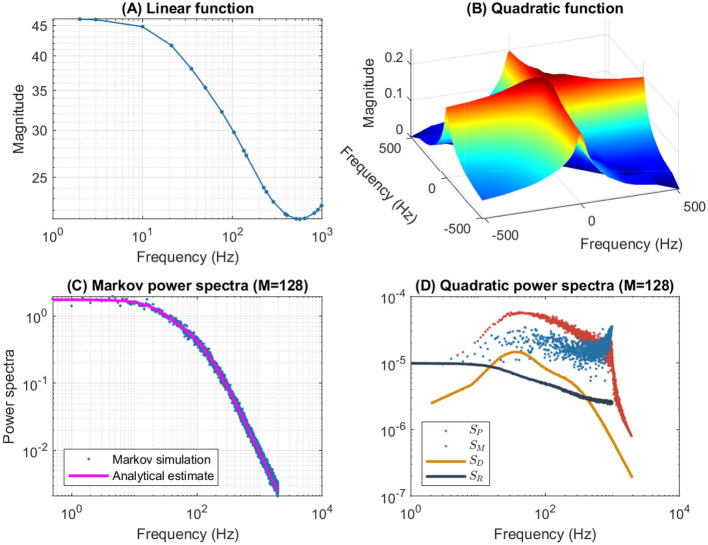
Frequency analysis of the *p*_2_ model for a 55 mV depolarization. The rate constants have been manually selected to approximate the frequency domain responses of the *n*^4^ model, which can be compared to Figure 6. **(A)** Linear function. **(B)** Quadratic function. **(C)** Markov power spectra (*M* = 128). **(D)** Quadratic power spectra (*M* = 128).

Figures 3C, 7 show an example of a *p*_2_ model, in which the rate constants have been manually selected to approximate the frequency domain responses of the *n*^4^ model, for a depolarization of 55 mV. As expected, both models show similar resonance behavior for the linear and quadratic responses. However, there are quantitative differences. First, the *S*_*D*_ quadratic power spectra of the *p*_2_ model (Figure 7) show not one, but two resonances reflected by a peak and bump, unlike the *n*^4^ model (Figure 6). Second, the Markov fluctuations of the *p*_2_ model (Figure 3C) are also different with the indication of more than one time constant illustrated by the inflection just before the final slope of *S*_2_, unlike the *n*^4^ model (Figure 2C) for which *S*_1_ fits *S*_IK_ accurately.

**Figure 8:**
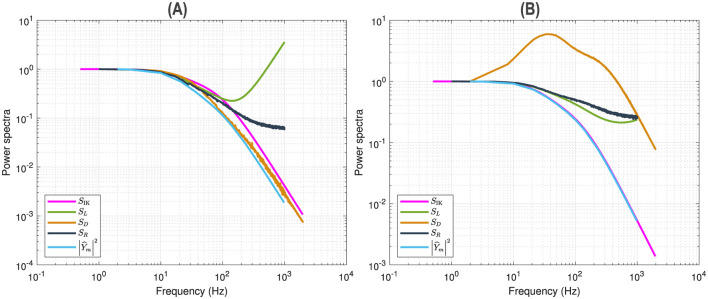
Power spectral analysis of the *p*_2_ model. Comparison between the analytical estimate of Markov fluctuations *S*_IK_ (magenta), the linear power spectrum *S*_*L*_ (green), the quadratic power spectra *S*_*D*_ (orange) and *S*_*R*_ (black), the modified squared admittance $|{Ŷ}_{m}{|}^{2}$ (blue). **(A)** Simulations for a 5 mV depolarization. **(B)** Simulations for a 55 mV depolarization.

Figure 8B shows the superposition of five curves of the *p*_2_ model for a 55 mV depolarization, using the same presentation as for the *n*^4^ model in Figure 5B. As with the *n*^4^ model, the modified squared admittance $|{Ŷ}_{m}{|}^{2}$ nearly superimposes on the Markov fluctuations *S*_IK_. However, the power spectra are quite different between the two models. The inflection of the Markov fluctuations *S*_IK_ is matched by the modified squared admittance $|{Ŷ}_{m}{|}^{2}$, which is consistent with the fact that the analytical expressions for Markov fluctuations and admittance both have two relaxation processes in the *p*_2_ model. In contrast, the *n*^4^ model has only one relaxation process due to the linearization of the exponentiation. Similarly, the modified squared admittance $|{Ŷ}_{m}{|}^{2}$ agrees well, at low frequencies, with the linear power spectrum *S*_*L*_ and column-mean-square component *S*_*R*_. Interestingly, the frequency doubling component *S*_*D*_ exhibits a double resonance, suggesting the existence of more than one relaxation process.

#### 3.7.2 Depolarization of 5 mV

**Figure 9:**
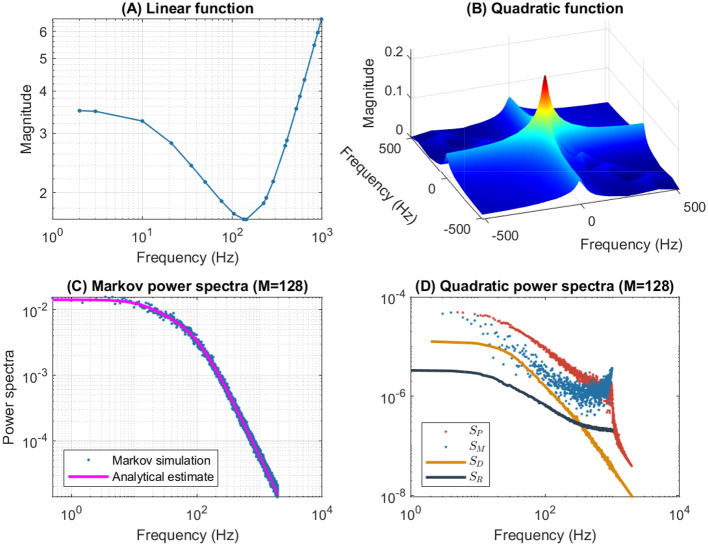
Frequency analysis of the *p*_2_ model for a 5 mV depolarization. The rate constants have been manually selected to approximate the frequency domain responses of the *n*^4^ model, which can be compared to Figure 4. **(A)** Linear function. **(B)** Quadratic function. **(C)** Markov power spectra (*M* = 128). **(D)** Quadratic power spectra (*M* = 128).

Figure 9 shows the same simulations as in Figure 7 but for a depolarization of 5 mV. The results are quite similar to the *n*^4^ model in Figure 4. The upper left plot shows a typical low frequency linear admittance anti-resonance. The upper right plot shows the QSA matrix as a 3D representation, which shows no anti-resonance. The lower left plot shows the power spectra of the Markov simulation superimposed on the analytical estimate, both of which reveal the characteristics of a low-pass filter. The lower right plot shows the quadratic power spectra averaged over several ODE simulations, all of these responses exhibit low-pass filter characteristics, but they flatten out by reaching a constant value at high frequencies, with the exception of *S*_*D*_(2|ω_*k*_|) at frequency doubling for which the amplitudes decrease at high frequencies.

Figure 8A shows that, as with the *n*^4^ model at 5 mV in Figure 5A, the linear behavior represented by the modified squared admittance $|{Ŷ}_{m}{|}^{2}$ does not accurately describe quadratic power spectra or Markov fluctuations. In particular, as with the *n*^4^ model at 5 mV, the modified squared admittance and quadratic power spectra of the *p*_2_ model have lower frequency components than the Markov fluctuations.

## 3.8 Validity

Depolarizations at 5 and 55 mV were selected to investigate potassium channel dynamics under contrasting conditions for both the *n*^4^ model and the *p*_2_ model. At 5 mV, $n_{\infty }^{4}≃0.0247$ and $p_{2}^{\infty }≃0.0297$ indicate negligible activation, reflecting the near-closed state of the channels. At 55 mV, $n_{\infty }^{4}≃0.596$ and $p_{2}^{\infty }≃0.565$ correspond to partial activation, illustrating the dynamics of channel opening under significant depolarization. These voltage levels were chosen to explore and compare the behavior of the two models across a physiologically relevant range under identical stimulation conditions.

## 4 Discussion

### 4.1 Spontaneous fluctuations and nonlinear responses

Neuronal fluctuations influence both transient and steady state responses, which fundamentally set the thresholds for neuronal impulse propagation. The fluctuating behavior of neurons in the central nervous system can be divided into three categories: (1) subthreshold synaptic bombardment from multiple networks, (2) additional nonlinear components generated for a particular stimulus, and (3) intrinsic spontaneous noise independent of external stimulus. Computational neuroscience is an attempt to understand the network behavior of the nervous system. However, it is highly dependent on neuronal models determined from numerous systems using very restricted data sets. The tools for extracting experimental data are impressive, ranging from current and voltage clamp, single channel and noise analysis, multi-electrode arrays recording activity on a population of cells, imaging techniques to estimate activity in intact systems, transfer function analysis and a variety of nonlinear approaches such as Wiener and Volterra kernels. The QSA method used in this article refines the quantitative characteristics of neuronal models in the frequency domain.

The above simulations shown for the potassium conductance suggest that the non-smooth fluctuations of the QSA power spectra *S*_*P*_ and *S*_*M*_ generate an alternative kind of noise due to the complexity of nonlinear interactions, which is not identical to the spontaneous fluctuations simulated by a stochastic Markov process. Although each individual QSA characterization uses a limited number of non-overlapping frequencies, this is overcome relatively well by averaging the power spectra for different stimulus sets. The *p*_2_ sequential model of the ion channel suggests that the fluctuation-dissipation theorem may be, in part, valid since both the linear and fluctuation spectra have similar relaxation times (corner frequencies), which is not the case for the *n*^4^ model.

QSA analysis of a Markov model requires the averaging of individual trace measurements using the same multi-sinusoidal stimulus, which will converge to the deterministic response if a sufficient number of averages are performed. QSA analysis of individual trace measurements leads to highly variable QSA power spectra, unlike the same procedures performed on deterministic models, which are invariant. As real biological neuronal cells are intrinsically fluctuating, QSA experiments on patch clamped neurons have been performed on averaged trace measurements (Magnani and Moore, 2011), leading to a deterministic response that averages out the spontaneous fluctuation behavior of ion channels. The above-mentioned QSA power spectra were performed with random QSA stimulus frequencies applied to ODEs in order to compare them with the Markov noise power spectra. Alternatively, a single set of QSA stimulus frequencies can be applied to a set of Markov simulations, in which case the power spectra of the QSA matrix coefficients for the same stimulus can be averaged to determine how prominent the Markov model's responses are at the nonlinear interactive frequencies. This can be called an average QSA Markov noise power spectrum.

**Figure 10:**
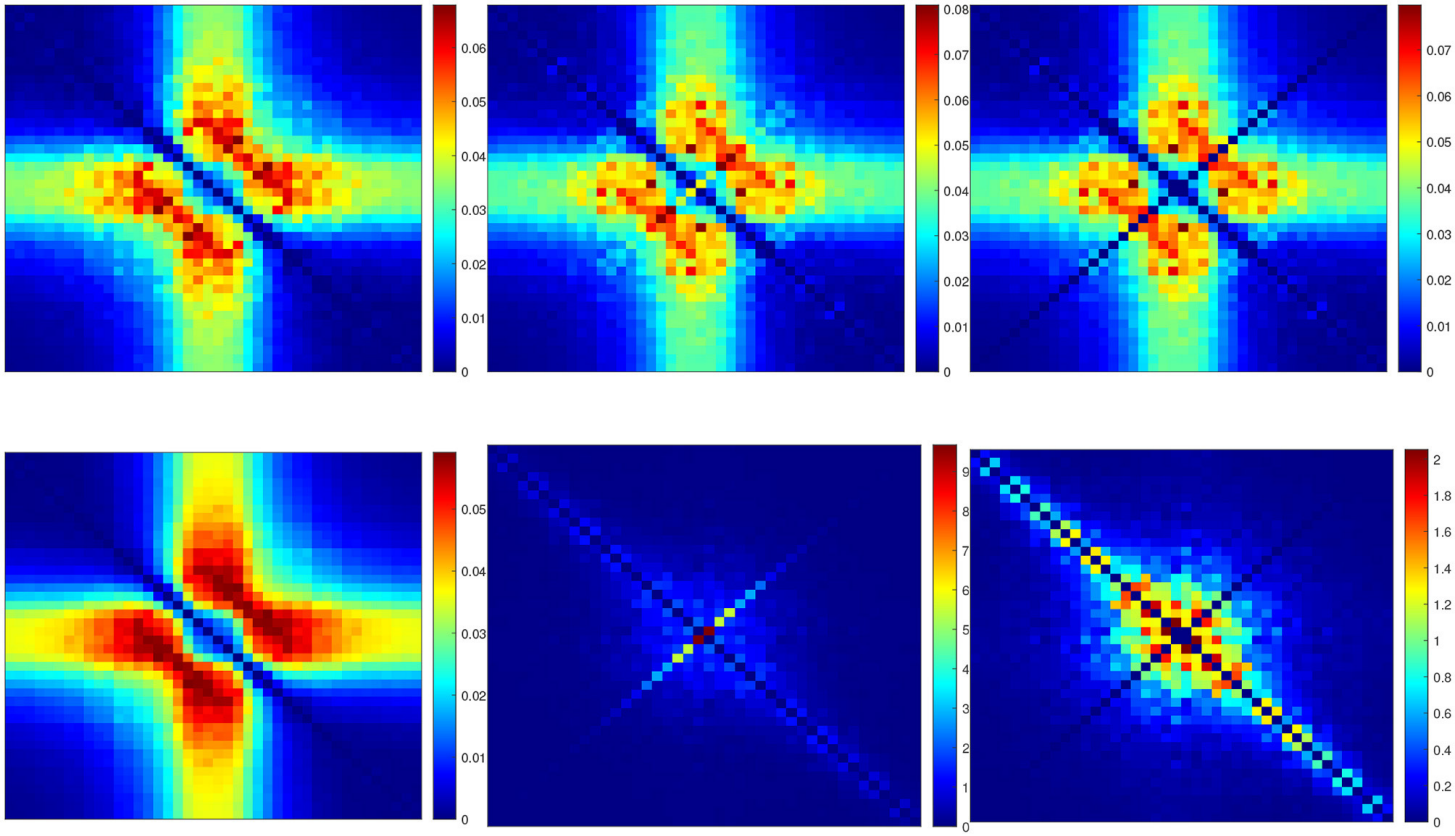
Power spectra of the QSA matrix coefficients of the *p*_2_ model for a 55 mV depolarization. **(Upper row)** Stimulus amplitude 4 mV. **(Lower row)** Stimulus amplitude 1 mV. **(Left column)** Deterministic ODE equations. **(Middle column)** QSA analysis performed on each individual Markov simulation for 16 iterations. **(Right column)** Identical to the middle column but with the diagonal (harmonic frequencies) removed (set to zero).

Figure 10 shows the power spectra of the QSA matrix coefficients of the *p*_2_ model for a 55 mV depolarization, comparing two different stimulus amplitudes 4 mV (top) and 1 mV (bottom). Stimulus amplitudes are relatively larger than those used previously in deterministic analyses, as quadratic responses must overcome spontaneous fluctuations, otherwise the quality of noisy signals is too degraded. The two plots on the left column represent the power spectra of the usual deterministic ODE equations, which serve as a reference. As expected, they are similar at 4 and 1 mV. The two plots in the middle column represent the power spectra of the QSA Markov noise (QSA analysis performed on each individual Markov simulation). Surprisingly, the result is similar to that of ODE at 4 mV, but dramatically different at 1 mV. In particular, the enhanced diagonal follows harmonic frequencies. The right column shows the same power spectra as the middle column, but with the diagonal (harmonic frequencies) removed (set to zero). The result remains dramatically different from the ODE at 1 mV, which means that both the diagonal and cross-terms responses represent another quadratic function. Namely, spontaneous Markov fluctuations modify the neuronal quadratic function as the stimulus amplitude decreases. It is interesting to note that harmonic frequencies of the QSA Markov noise are also enhanced at 4 mV, but the matrix is essentially similar to that of ODE. The enhanced diagonal may be due to the fact that the modified squared admittance $|{Ŷ}_{m}{|}^{2}$ predicts the Markov fluctuations *S*_IK_ at a 55 mV depolarization, as shown in Figure 8B. Each squared frequency component generates frequency doubling. Thus, Markov fluctuations contribute to the harmonic frequencies in the QSA analysis of each individual Markov simulation, as well as at, to a lesser extent, to the interactive frequencies since the Markov noise cloud is not a smooth curve.

**Figure 11:**
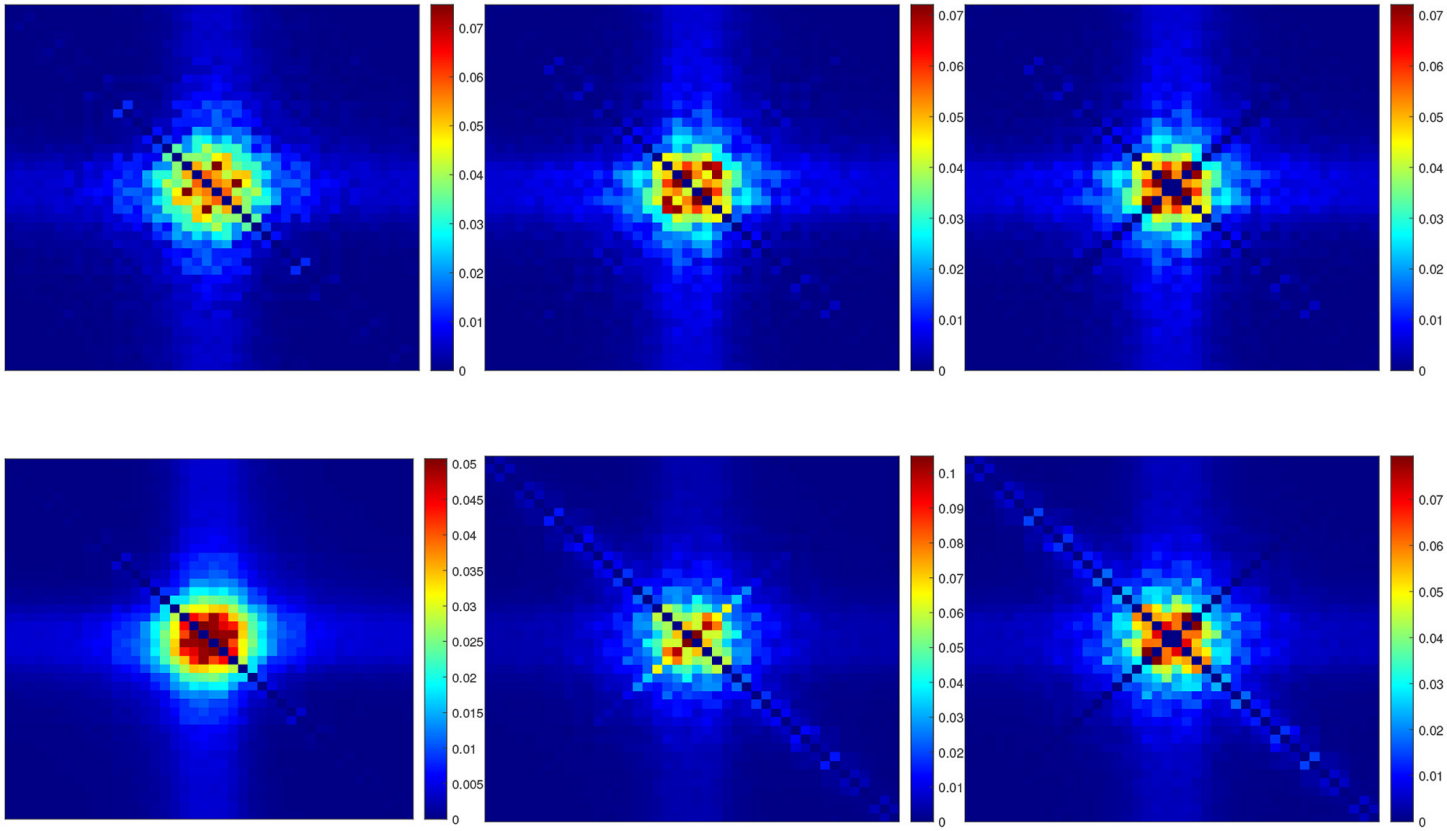
Power spectra of the QSA matrix coefficients of the *p*_2_ model for a 5 mV depolarization. **(Upper row)** Stimulus amplitude 4 mV. **(Lower row)** Stimulus amplitude 1 mV. **(Left column)** Deterministic ODE equations. **(Middle column)** QSA analysis performed on each individual Markov simulation for 16 iterations. **(Right column)** Identical to the middle column but with the diagonal (harmonic frequencies) removed (set to zero).

Figure 11 supports this hypothesis as illustrated with the power spectra of the QSA matrix coefficients of the *p*_2_ model for a 5 mV depolarization, comparing two different stimulus amplitudes 4 mV (top) and 1 mV (bottom). At this low depolarization level, the modified squared admittance $|{Ŷ}_{m}{|}^{2}$ no longer predicts the Markov fluctuations *S*_IK_, as shown in Figure 8A. In this case, the diagonal of the QSA Markov noise is smaller and the power spectra of the QSA matrix coefficients are similar to those of the ODE at 4 mV and 1 mV. Nevertheless, the diagonal remains somewhat enhanced in the QSA Markov noise, likely because Markov fluctuations and modified squared admittance $|{Ŷ}_{m}{|}^{2}$ have a similar appearance.

**Figure 12:**
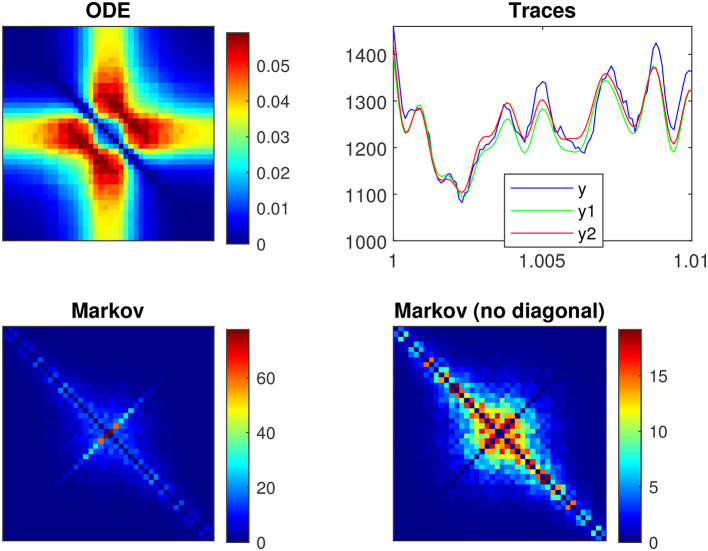
Power spectra of the QSA matrix coefficients of the *p*_2_ model for a membrane surface area $A_{\text{K}}=50\text{μ}{\text{m}}^{2}$, a 55 mV depolarization and a stimulus amplitude 1 mV. **(Top left)** Deterministic ODE equations. **(Top right)** A single trace (blue curve) from Markov simulations in time domain (s, pA) with linear (green curve) and quadratic (red curve) analyses. **(Bottom left)** QSA analysis performed on each individual Markov simulation for 16 iterations. **(Bottom right)** Identical to the bottom left but with the diagonal (harmonic frequencies) removed (set to zero).

**Figure 13:**
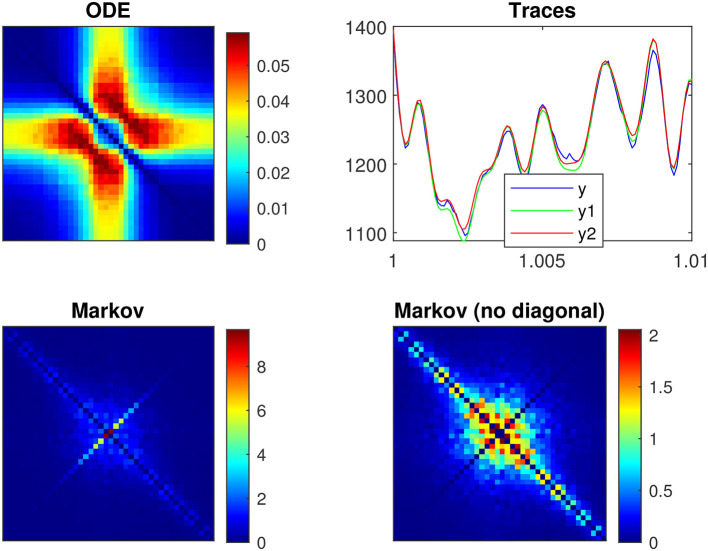
Power spectra of the QSA matrix coefficients of the *p*_2_ model for a membrane surface area $A_{\text{K}}=500\text{μ}{\text{m}}^{2}$, a 55 mV depolarization and a stimulus amplitude 1 mV. The plots were generated using the same presentation as in Figure 12.

**Figure 14:**
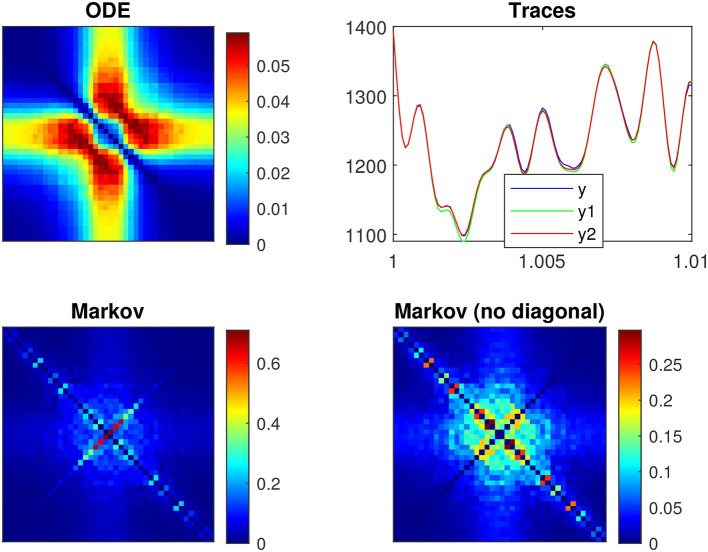
Power spectra of the QSA matrix coefficients of the *p*_2_ model for a membrane surface area $A_{\text{K}}=5,000\text{μ}{\text{m}}^{2}$, a 55 mV depolarization and a stimulus amplitude 1 mV. The plots were generated using the same presentation as in Figure 12.

**Figure 15:**
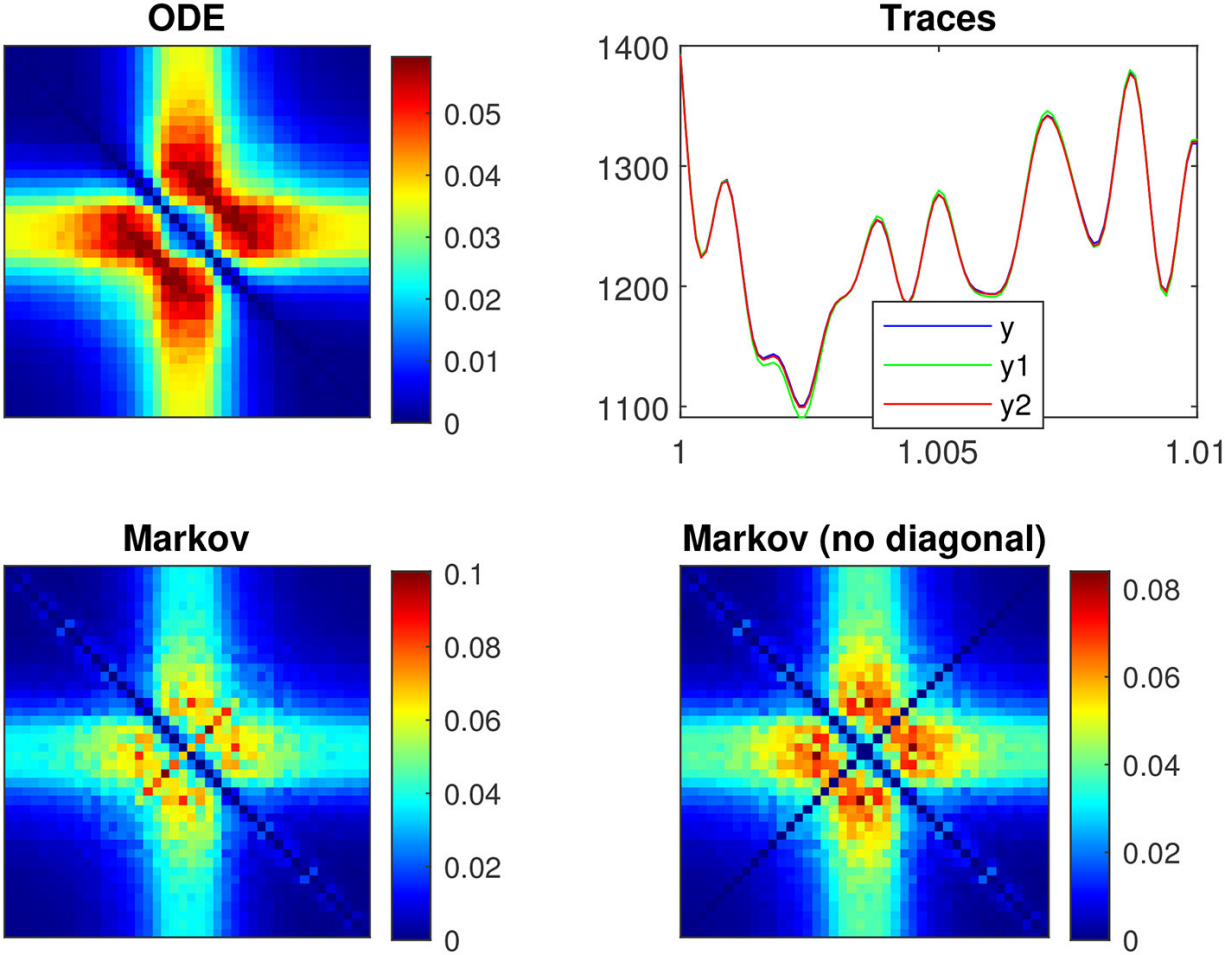
Power spectra of the QSA matrix coefficients of the *p*_2_ model for a membrane surface area $A_{\text{K}}=50,000\text{μ}{\text{m}}^{2}$, a 55 mV depolarization and a stimulus amplitude 1 mV. The plots were generated using the same presentation as in Figure 12.

Figures 12–15 reinforce this interpretation by varying the surface area of the membrane of the *p*_2_ model for a 55 mV depolarization and stimulus amplitude 1 mV. The larger the surface area of the membrane, the lower the noise effect. The smallest areas $A_{\text{K}}=50\text{μ}{\text{m}}^{2}$, $A_{\text{K}}=500\text{μ}{\text{m}}^{2}$, $A_{\text{K}}=5,000\text{μ}{\text{m}}^{2}$ show a behavior similar to Figure 10, that is to say spontaneous Markov fluctuations modify the neuronal quadratic function. However, the largest area $A_{\text{K}}=50,000\text{μ}{\text{m}}^{2}$ shows a behavior that partially recovers the ODE and better without the diagonal. This suggests that increasing the stimulus amplitude or decreasing the noise amplitude have similar effects on the quadratic function expressed. However, the two approaches are not equivalent since the amplitude of the stimulus tends to be modulated by the inputs of a neuron while the amplitude of the noise depends on the anatomy of a neuron.

This suggests that responses to neuronal stimuli would be different for quiet versus noisy neurons. Quiet neurons would exhibit complex nonlinear frequency responses, whereas the nonlinear responses of noisy neurons would tend to have additional frequency components, particularly at harmonic frequencies of the stimulus. Thus, individual neurons within a neuronal network would process input stimuli according to background synaptic activity, which is likely to be the main determinant of neuronal noise.

### 4.2 Potential applications

The spectral analysis of spontaneous fluctuations and nonlinear responses in ion channels, particularly potassium channels, provides an effective approach for understanding fundamental mechanisms of neuronal dynamics. These methods, which combine computational modeling and data analysis, are closely aligned with the principles of neuroinformatics by enabling the systematic interpretation of biological signals. This methodology suggests practical applications in areas relevant to real neuroinformatics scenarios.

The development of computational models of neurons provides a natural platform for applying spectral analysis, offering a way to connect ion channel dynamics with broader neuronal behavior. For example, Martin and Pedersen (2024) investigates the critical roles of HCN and M-type potassium channels in modeling and analyzing cAMP-induced mixed-mode oscillations in cortical neurons. Their use of a Hodgkin–Huxley-type model presents an opportunity to apply spectral analysis methods to characterize the behavior of different types of potassium channels in the context of cortical neurons.

The study by Pettersen et al. (2014) investigates power spectral densities (PSDs) of the soma potential, soma membrane current, and the single neuron contribution to the electroencephalogram (EEG). Their analytical approach, based on linear neuronal cable theory, explores how power laws may arise from the intrinsic biophysical properties of single neurons, independent of complex network interactions. Integrating spectral analysis methods within this theoretical basis could help clarify the interplay between ion channel dynamics and macroscopic neuronal activity patterns. Such integration holds potential for applications requiring accurate modeling of neuronal behavior, such as in EEG analysis or brain-computer interfaces.

Traumatic brain injuries and repetitive head impacts can result in altered neuronal excitability, with potassium channels playing an important role. Chapman et al. (2023) developed an *in silico* model of mouse CA1 pyramidal cells to identify ion channels contributing to hypoexcitability in this context. The model used morphology from the neuromorpho archive (Hines, 2009) and simulations performed with the NEURON platform (Ascoli et al., 2007). Applying spectral analysis methods to such realistic models could help identify spectral signatures of potassium channel dysfunction. Such analyses align with the goals of precision medicine and targeted therapeutic strategies.

Operator learning provides effective tools for modeling the complex dynamics of neurons. Centofanti et al. (2024) demonstrated the potential of operator learning, including Deep Operator Networks (DeepONets), Fourier Neural Operators (FNOs), and Wavelet Neural Operators (WNOs), to model the Hodgkin–Huxley system. Using square pulse stimuli, these methods map input currents to transmembrane potentials, capturing the dynamics of the Hodgkin–Huxley model. The square pulse stimuli could be extended to QSA multi-sinusoidal inputs to more precisely probe subthreshold nonlinear responses, although this may require some methodological adjustments. Extending their approach to a stochastic Markov model appears to be another challenge. Building on these ideas, spectral analysis methods could be combined with operator learning approaches to support applications in neuroscience and AI.

The principles of quantum mechanics provide innovative tools for understanding and modeling neuronal dynamics. The quantized Hodgkin–Huxley model proposed by Gonzalez-Raya et al. (2019) associates potassium conductance with a memristor driven by sinusoidal currents. Extending this method with QSA multi-sinusoidal driving could broaden its scope by probing nonlinear interactions across multiple frequencies. Additionally, Bradley et al. (2020) describe the use of quantum states to represent classical probability distributions over sets of sequences. Their use of a density operator to encode probabilistic information offers a potential approach to compare stochastic spontaneous fluctuations to nonlinear responses encoded in the QSA matrix, which is Hermitian. Together, these concepts offer promising directions for exploring quantum-inspired models of neuronal dynamics.

### 4.3 Comparing the *p*_2_ model with existing approaches

The *p*_2_ model is constructed as a variant of the *n*^4^ five-state Markov chain with two independent rate constants, formulated as a three-state Markov chain and generalized to four independent rate constants. In this way, the *p*_2_ model avoids explicit exponentiation, yet it can represent the minimal degree of nonlinearity *n*^2^ as well as models without exponentiation. Moreover, the rate constants of the *p*_2_ model can be tuned to preserve the key dynamical features of the *n*^4^ model, as evaluated through spectral analysis methods.

The relevance of decreasing the degree of exponentiation to *n*^2^ for potassium permeability is exemplified by the work of Frankenhaeuser and Huxley (1964) in their study of myelinated nerve fibers. This adaptation, motivated by experimental observations and computational simplicity, preserved the essential dynamical behaviors. Similarly, the *p*_2_ model generalizes this approach by providing a flexible three-state Markov representation with independent rate constants.

The master equation of the *p*_2_ model given in Equation 8 is consistent with established methods in the literature. For example, Güler (2015) describes a similar master equation in the context of the minimal diffusion formulation of Markov chain ensembles and its application to ion channel clusters. This connection highlights the relevance of such master equations in modeling ion channel dynamics and their stochastic behaviors.

Another example illustrating a similarity with the *p*_2_ model formulation is provided by Schmandt and Galán (2012), who developed the stochastic-shielding approximation of Markov chains to efficiently simulate random ion-channel gating. They describe a three-state Markov chain and use variables *N*_*k*_(*t*) and *N*_*ij*_(*t*), representing the number of elements in state *k* and the number transitioning from state *i* to *j*, respectively, similar to the variables described in the above section on stochastic Markov simulations.

An insightful study is provided by Güler (2013), which investigates the impact of introducing noise into rate constants in Hodgkin–Huxley models. This approach modifies the standard rate constants α and β with stochastic terms, while the *p*_2_ model applies fixed multiplicative adjustments to these rates. This work could inspire a potential extension of the *p*_2_ model to incorporate stochastic variability in the rate constants.

### 4.4 Experimental considerations

The simulations and theoretical analyses presented in this article involve two key aspects for experimental validation: the deterministic QSA method and stochastic Markov models, both of which provide complementary insights into neuronal dynamics.

The QSA method was specifically designed to support both model-based and experimental measurements. In particular, the multi-sinusoidal stimuli are carefully generated to avoid frequency overlap at first and second orders, enabling a direct evaluation of the QSA matrix from input-output data. In this article, the QSA method has been extended by averaging multiple measurements to improve the accuracy of the power spectra. However, each individual measurement remains the response to a stimulus without overlap, ensuring that this extension does not alter the original experimental protocol. Consequently, the spectral analyses of linear and quadratic responses described in the above sections could potentially be applied to experimental measurements. Importantly, the QSA method has already been validated using experimental data from whole-cell patch-clamp recordings of prepositus hypoglossi nucleus neurons (Magnani and Moore, 2011; Magnani et al., 2013) and medial entorhinal cortex neurons (Magnani et al., 2014). Furthermore, these validated data were compared with conductance-based models of the corresponding neurons, emphasizing the relevance of the method for bridging experimental and theoretical approaches.

The validation of stochastic Markov models, such as the *n*^4^ and *p*_2_ models, for describing ion channel kinetics presents a significant experimental challenge, as it inherently involves solving an inverse channel–fitting problem (Cannon and D'Alessandro, 2006). From a biophysical perspective, Markov models are considered more flexible than the Hodgkin–Huxley model because they incorporate discrete states that better capture the complexity of voltage-gated ion channels. For example, Andreozzi et al. (2019) compared Hodgkin–Huxley and Markov models for a sodium channel (Na_V_1.5) with experimental data, indicating limitations of the Hodgkin–Huxley model and providing simplified Markov models as effective tools to approximate the complexity of ion channel kinetics.

The *n*^4^ exponentiation in the Hodgkin–Huxley model is commonly interpreted as describing a potassium ion channel composed of four independent gates. If each gate is open with probability *n*, the channel as a whole is open with probability *n*^4^. However, gating independence is merely an assumption. This nuance is addressed by Mannuzzu and Isacoff (2000), who show that the late gating steps of Shaker potassium channels involve cooperative interactions between subunits. Consistently, the *p*_2_ model does not rely on the concept of independent gates. Instead, it relies on independent rate constants. This approach allows the *p*_2_ model to represent behaviors such as *n*^2^, as well as variants without explicit exponentiation. Moreover, this article has demonstrated that the *p*_2_ and *n*^4^ models can produce similar neuronal responses, suggesting that the underlying kinetic schemes are more essential than gating independence for capturing channel behavior.

The *p*_2_ model (or an extension) could potentially be further adapted to approximate experimental data as demonstrated here theoretically to approximate the *n*^4^ model. Power spectra could be compared to investigate discrepancies between fluctuation and QSA measurements (linear, quadratic). Earlier work by Fishman et al. (1983) compared fluctuation power spectra and linear admittance measurements of sodium current kinetics in squid axons, demonstrating significant discrepancies between the two methods and highlighting the nonlinear nature of microscopic sodium channel kinetics. This approach could shed light on the dynamics of ion channels and contribute to the validation of theoretical models.

Molecular dynamics simulations offer a promising approach to improve Markov models. Catacuzzeno et al. (2024) proposed an innovative method to build Markov models of ion channel permeation from molecular dynamics simulations. Combining these simulations with spectral analysis methods and the *p*_2_ model remains a complex challenge but holds potential to achieve a more accurate representation of ion channel behavior under physiological conditions.

### 4.5 Implications for neuromorphic systems

Neuromorphic systems represent an innovative approach in neuroinformatics, based on biologically inspired principles to mimic dynamic processes of neuronal systems. By leveraging non-von Neumann architectures, they enable efficient modeling of complex neuronal functions. The potential of these systems has been demonstrated in various contexts: Sun et al. (2020) explored hippocampal dynamics using scalable digital neuromorphic models; Yang et al. (2021a) developed BiCoSS, a platform integrating multigranular spiking networks for advanced cognitive tasks; Yang et al. (2021b) applied neuromorphic principles to cerebellar motor learning. These studies collectively highlight the ability of neuromorphic systems to bridge biological relevance with computational efficiency, contributing to a deeper understanding of neuronal mechanisms.

The inherent complexity of neuronal systems, particularly the nonlinear dynamics of voltage-gated ion channels, poses significant challenges for neuromorphic systems, which must balance biological realism with computational efficiency. Neuronal processing is not limited to high-level brain functions but also encompasses computations at the level of individual neurons and voltage-gated ion channels, as characterized in this article through spectral methods such as linear impedance, quadratic interactions (QSA matrix), and microscopic noise (power spectra), which means that simplifying while preserving key aspects of neuronal computations in neuromorphic systems is a delicate task. For example, Sun et al. (2020) used the Izhikevich model (Izhikevich, 2003) in their neuromorphic implementation of hippocampal networks, achieving computational efficiency while simplifying the underlying biophysical complexity of ion channels. As pointed out in Yang et al. (2018), the Izhikevich model, while computationally efficient, does not fully capture the full range of ionic conductance dynamics, which may limit its use in studies requiring high biological accuracy. However, platforms such as BiCoSS (Yang et al., 2021a) demonstrate how neuromorphic systems can enhance biological representation and offer flexibility to address such challenges.

Biological neurons have highly specific properties, which present another significant challenge for neuromorphic systems to replicate their dynamics. As explained by Idoux et al. (2008), the prepositus hypoglossi nucleus (PHN) contains distinct neuronal populations, such as type B and type D neurons, which differ in properties like action potential shape and oscillatory behavior, highlighting the diversity within a single brain region. These neurons also exhibit varying electrotonic lengths: type B neurons are more compact, allowing distal dendritic inputs to significantly influence somatic activity, whereas type D neurons have extended electrotonic lengths, emphasizing the computational role of dendrites. Furthermore, the persistent sodium conductance (*g*_NaP_), present in all PHN neurons, modulates activity differently depending on its somatic or dendritic localization, further illustrating the finely tuned specificity of neuronal mechanisms. Emulating multicompartment neurons with dendritic computations is essential for advancing the biological realism of neuromorphic systems, as highlighted by Yang et al. (2019). The platform BiCoSS (Yang et al., 2021a) supports multi-level, multigranule computing, including both point neuron and compartmental neuron. Furthermore, Beaubois et al. (2024) explored multicompartment Hodgkin–Huxley models in neuromorphic applications to better capture the complex morphological and functional properties of neurons.

Addressing the duality between membrane potential activity (continuous, analog) and spiking activity (discrete, event-driven) remains a significant challenge for neuromorphic systems, as these processes are intrinsically linked yet distinct in neuronal computation. Several methods have been developed to explore this duality. For example, Ris et al. (2001) showed that neurons in the medial vestibular nucleus exhibit resonance in the modulation of spike discharge, where spiking activity is closely tied to underlying membrane potential dynamics. Moreover, Recio-Spinoso et al. (2005) demonstrated that second-order Wiener kernels applied to spiking activity in auditory nerve fibers capture nonlinear interactions and temporal dynamics that first-order analyses cannot, providing deeper insights into the relationship between input sound stimuli and neuronal output. In another approach, Magnani et al. (2014) used quadratic sinusoidal analysis (QSA) in neurons of the medial entorhinal cortex to study how subthreshold nonlinearities influence spiking responses, highlighting the intricate link between membrane potential dynamics and suprathreshold activity. Additionally, Inoue et al. (2021) proposed a data-driven method to estimate latent variables and parameters of the Izhikevich neuron model using only spike-train data, employing the replica exchange particle-Gibbs with ancestor sampling (REPGAS) method. These methods are useful tools for understanding the interplay between subthreshold and suprathreshold dynamics and could provide insights for enhancing the biological realism of neuromorphic systems, as explored in Sun et al. (2020) and Yang et al. (2021a,b).

Maintaining eye movement stability in response to head or visual field movements relies on the precise coordination of neural mechanisms that integrate sensory inputs and sustain motor commands. Yang et al. (2021b) examined the optokinetic reflex (OKR) in the context of a neuromorphic cerebellar model, exploring how their cerebellar-inspired architecture adapts to retinal slip signals through mechanisms of supervised motor learning and synaptic plasticity. These mechanisms were applied in simulations of OKR adaptability to replicate dynamic responses like gain modulation and temporal synchronization in eye movement control. Building on biological insights, Magnani et al. (2013) investigated prepositus hypoglossi nucleus neurons, essential for maintaining eye position by integrating head velocity signals, highlighting the role of nonlinear dynamics driven by persistent sodium conductance (*g*_NaP_) and active dendritic structures for sustaining the effects needed for gaze stabilization. Furthermore, Koulakov et al. (2002) introduced a robust model of the neural integrator based on bistable units, demonstrating how stability in eye position control can be achieved without fine-tuning of recurrent synaptic weights, which may be particularly relevant for neuromorphic systems. These studies show the importance of voltage-gated mechanisms and individual neuron dynamics in achieving eye movement stability, which have potential relevance for the design of neuromorphic systems that aim to replicate complex sensory-motor coordination.

Grid cells in the medial entorhinal cortex play a central role in spatial representation and navigation, providing a coordinate-like system to encode the position of an organism within its environment. These circuits are of particular relevance for neuromorphic systems due to their role in modeling spatial and cognitive functions. Krishna et al. (2021) represents a significant advancement in the hardware implementation of biologically inspired spatial navigation systems, presenting a biomimetic FPGA-based model that combines grid cells and place cells. However, the model abstracts away the biophysical details of individual neurons, like voltage-gated ion conductances, which are crucial for capturing the temporal dynamics of neurons, such as phase precession and resonance properties. From a biological perspective, Magnani et al. (2014) investigated the nonlinear properties of layer II stellate neurons in the rat medial entorhinal cortex (MEC) using quadratic sinusoidal analysis (QSA). The study revealed how dendritic filtering shapes somatic nonlinearities, with responses strongly influenced by the frequency content of stimuli. Persistent sodium conductance (*g*_NaP_) was identified as a key source of nonlinearity, and near-threshold linear and nonlinear responses reliably predicted suprathreshold behavior, including action potential modulation. These findings emphasize the importance of biologically realistic modeling to connect single neuron voltage-gated mechanisms with scalable implementations of spatial navigation circuits. Thus, multigranular platforms like BiCoSS (Yang et al., 2021a) show particular promise for integrating such biophysical details into neuromorphic designs.

## 5 Conclusion

In conclusion, neurons clearly have linear and nonlinear responses to input stimuli. Linear responses are not just mirror images of the stimulus, they are clearly capable of enhancing certain frequencies due to linear resonance behavior. The linear response of resonance for the Hodgkin–Huxley potassium conductance is only present if the steady state value of *n* is voltage dependent, namely $\frac{dn_{\infty }}{dV_{0}}>0$, i.e. the resonance is a linear response that depends on the nonlinear property of the conductance. In general, nonlinear responses are considerably more complex, as shown by the presence of new interactive frequencies in the neuronal response that are not present in the input signal. The fluctuations present in neurons are due to voltage-dependent random mechanisms for which probabilities are controlled both linearly and nonlinearly and, as suggested above, ongoing synaptic activity can alter the nature of nonlinear responses (in the sense that QSA matrix can reflect fluctuations for small stimulus amplitudes).

## Data Availability

The raw simulation data supporting the conclusions of this article will be made available by the authors, without undue reservation.
